# The ADAR1 editome reveals drivers of editing-specificity for ADAR1-isoforms

**DOI:** 10.1093/nar/gkad265

**Published:** 2023-04-07

**Authors:** Renata Kleinova, Vinod Rajendra, Alina F Leuchtenberger, Claudio Lo Giudice, Cornelia Vesely, Utkarsh Kapoor, Andrea Tanzer, Sophia Derdak, Ernesto Picardi, Michael F Jantsch

**Affiliations:** Center for Anatomy and Cell Biology, Division of Cell and Developmental Biology, Medical University of Vienna, Schwarzspanierstrasse 17, A-1090 Vienna, Austria; Center for Anatomy and Cell Biology, Division of Cell and Developmental Biology, Medical University of Vienna, Schwarzspanierstrasse 17, A-1090 Vienna, Austria; Center for Integrative Bioinformatics Vienna (CIBIV) Max Perutz Labs, University of Vienna and Medical University of Vienna, Campus Vienna Biocenter 5, A-1030 Vienna, Austria; Department of Bioscience, Biotechnology and Biopharmaceutics, University of Bari Aldo Moro, University Campus “Ernesto Quagliariello”, Via Orabona 4, Bari, Italy; Center for Anatomy and Cell Biology, Division of Cell and Developmental Biology, Medical University of Vienna, Schwarzspanierstrasse 17, A-1090 Vienna, Austria; Center for Anatomy and Cell Biology, Division of Cell and Developmental Biology, Medical University of Vienna, Schwarzspanierstrasse 17, A-1090 Vienna, Austria; Center for Anatomy and Cell Biology, Division of Cell and Developmental Biology, Medical University of Vienna, Schwarzspanierstrasse 17, A-1090 Vienna, Austria; Core Facilities Medical University of Vienna, Spitalgasse 23, A-1090 Vienna, Austria; Department of Bioscience, Biotechnology and Biopharmaceutics, University of Bari Aldo Moro, University Campus “Ernesto Quagliariello”, Via Orabona 4, Bari, Italy; Institute of Biomembranes and Bioenergetics (IBBE), National Research Council (CNR), Via Amendola 122, Bari, Italy; Center for Anatomy and Cell Biology, Division of Cell and Developmental Biology, Medical University of Vienna, Schwarzspanierstrasse 17, A-1090 Vienna, Austria

## Abstract

Adenosine deaminase acting on RNA ADAR1 promotes A-to-I conversion in double-stranded and structured RNAs. ADAR1 has two isoforms transcribed from different promoters: cytoplasmic ADAR1p150 is interferon-inducible while ADAR1p110 is constitutively expressed and primarily localized in the nucleus. Mutations in ADAR1 cause Aicardi – Goutières syndrome (AGS), a severe autoinflammatory disease associated with aberrant IFN production. In mice, deletion of ADAR1 or the p150 isoform leads to embryonic lethality driven by overexpression of interferon-stimulated genes. This phenotype is rescued by deletion of the cytoplasmic dsRNA-sensor MDA5 indicating that the p150 isoform is indispensable and cannot be rescued by ADAR1p110. Nevertheless, editing sites uniquely targeted by ADAR1p150 remain elusive. Here, by transfection of ADAR1 isoforms into ADAR-less mouse cells we detect isoform-specific editing patterns. Using mutated ADAR variants, we test how intracellular localization and the presence of a Z-DNA binding domain-α affect editing preferences. These data show that ZBDα only minimally contributes to p150 editing-specificity while isoform-specific editing is primarily directed by the intracellular localization of ADAR1 isoforms. Our study is complemented by RIP-seq on human cells ectopically expressing tagged-ADAR1 isoforms. Both datasets reveal enrichment of intronic editing and binding by ADAR1p110 while ADAR1p150 preferentially binds and edits 3’UTRs.

## INTRODUCTION

Adenosine to inosine deamination of RNA (A-to-I RNA editing) is a major nucleotide modification found in RNAs (1). In human and mouse transcriptomes, more than 10^6^ and 10^5^ A-to-I editing sites have been identified, respectively (2–4). A-to-I editing occurs in structured or double-stranded regions of RNA. The majority of editing sites is located in interspersed nuclear elements (SINEs) that are frequently located in introns and 3’UTRs (2,5). When two or more repeats are found in opposite orientation within an RNA, they tend to basepair and therefore can produce the double-stranded structures required for RNA-editing (6). Cellular machineries typically interpret inosines as guanosines. Therefore, RNA editing affects many cellular processes, including protein-coding, RNA-splicing, -folding and turnover (1,7).

In mammals, RNA editing is mediated by two catalytically active adenosine deaminases acting on RNA (ADARs): ADAR1 (*Adar*) and ADAR2 (*Adarb1*). ADARs contain a conserved deaminase domain and two or three dsRNA-binding domains that bind to dsRNA and highly structured RNA (Supplementary Figure S1) (8). ADAR1 is expressed from two different promoters giving rise to two isoforms: (i) interferon-inducible ADAR1p150 and (ii) constitutively expressed ADAR1p110 (9). Dysfunctions of ADAR1 can lead to Aicardi-Goutières syndrome (AGS), a fatal childhood encephalopathy accompanied with aberrant IFN signature (10,11). In mice, lack of ADAR1 leads to embryonic lethality by embryonic day E12.5 that is accompanied by IFN overproduction, hematopoietic failure, liver disintegration and widespread apoptosis (12,13). Mice bearing a catalytically inactive ADAR1^E861A^ knock-in mutation die at E13.5, which indicates that A-to-I editing is indeed the essential function of ADAR1 (14). Although the lack of ADAR2 is also not tolerated in mice and leads to postnatal lethality due to progressive seizures, the phenotype can be elegantly rescued by inserting a single point mutation mimicking editing in the glutamate receptor *Gria2* (15).

No specific ADAR1-substrate that could rescue the deleterious effect of ADAR1 dysfunction has been identified so far. Still, embryonic death of ADAR1-deficient mice can be rescued by concurrent deletion of the cytoplasmic dsRNA-sensor MDA5 or its downstream adaptor protein MAVS (14,16,17). Importantly, unique deletion of the ADAR1p150 isoform is embryonic lethal with IFN-overproduction. Like a full ADAR1 knockout, lack of ADAR1p150 can be rescued by concomitant deletion of MDA5 (17). In contrast, a specific knockout of ADAR1p110 is viable in mice, without an aberrant IFN signature (18).

Taken together, this shows that ADAR1p150–mediated editing is a specific and essential regulator of the MDA5-MAVS pathway and that ADAR1p110 alone cannot prevent an undesired innate immune response (17,18). ADAR1p150 contains a unique N-terminus bearing a Z-DNA binding domain α (ZBDα) and a nuclear export signal (NES) that mediates a prevalently cytoplasmic localization. In contrast, ADAR1p110 is localized to the nucleus. However, both isoforms can shuttle between the nucleus and the cytoplasm (19–22). ZBDα binds the unusual left-handed conformation of DNA and RNA (23). One of the most common ADAR1 mutations in AGS patients, P193A, lies in ZBDα. In AGS patients, the ADAR1^P193A^ allele exists in a compound-heterozygous stage with a second dysfunctional ADAR1 allele (11,24).

Interestingly, mice with mutations introduced in the ZBDα that extensively affect binding to nucleic acids in Z-conformation (N175A + Y179A, W197A) display reduced editing of SINE-containing RNAs. Moreover, these mice also develop a spontaneous MAVS-depended IFN-response (25–27). Still, mice carrying homozygous mutations P195A (mimicking human P193A) in ZBDα are phenotypically normal. Most interestingly, the human AGS phenotype is recapitulated once compound heterozygous mice are generated by introducing a copy of the ADAR1^P195A^ mutant allele together with a deletion of the second ADAR1 or ADAR1p150 allele. These mutant mice develop a severe disease that is driven by MDA5 (28). Considering the lack of phenotype in homozygous ADAR1^P195A^ mutant mice and the absence of AGS patients carrying homozygous ZBDα mutations it seems that this domain is not a main driver of AGS but rather contributes to the disease. Moreover, the contribution of ZBDα to ADAR1p150 editing-specificity and its impact on nuclear and cytoplasmic ADAR1p150-editing remain elusive. Recently, ZBDα has been shown to prevent activation of ZBP1 and thereby necroptosis (29–32). While the individual contribution of the different signaling pathways of ADAR1 are not yet entirely clear, these new and exciting manuscripts shed new light on the function of ZBDα as a sensor of Z-form nucleic acids.

In our study, we determine isoform-specific editing patterns in mouse cells and comprehensively analyze the ADAR1p150- and ADAR1p110-specific mouse-editome. Moreover, using mutated ADAR versions on a subset of substrates, we investigated the role of cytoplasmic localization and of ZBDα on ADAR1p150 editing-specificity. Our findings suggest that ZBDα has a minor contribution to editing specificity and that ADAR1p150 editing patterns are mostly driven by the cytoplasmic localization of this protein isoform. Additionally, we intersect our data with RIP-seq data on human cells to extensively examine ADAR1-isoform binding- and editing characteristics. While ADAR1p110 binds and edits intronic regions, ADAR1p150 shows specificity to exons and 3’UTRs.

## MATERIALS AND METHODS

### Cell culture and MEF generation

Human embryonic kidney 293 cells (HEK 293) were maintained in high-glucose Dulbecco's modified Eagle's medium (DMEM) (Thermo Fisher Scientific, Waltham, MA) supplied with 10% fetal bovine serum, pyruvate and l-glutamine.

### Generation and culture of mouse embryonic fibroblasts (MEFs)

11-day old embryos of genotype *Adar^−/−^; Adarb1^−/−^*; *Gria2^R/R^* were macerated and cells were cultured in DMEM supplemented with 20% FBS. Early passage MEFs were immortalized using lentiviral transduction (33).

### Plasmids

human ADAR1 plasmids were a kind gift of Mary O’Connell. cDNAs encoding ADAR1p150 or ADAR1p10 were tagged with a FLAG-tag at the amino terminus and a HIS-tag at the C-terminus (34). Plasmids were cloned into pcDNA3.1. Detection of ADARs was monitored by western blotting using an ADAR1 specific antibody, a FLAG-antibody or an anti HIS-antibody.

### Restoring ADAR1 expression in editing-deficient cells

0.7 × 10^6^ MEFs were electroporated with 5 μg or 10 μg plasmid DNA using a Neon Transfection System (Thermo Fisher Scientific, Waltham, MA) with parameters: 1350 V, 30 mS, 1 pulse and 100 μl neon tip.

### Library construction

Cells were harvested 24 h post-transfection. RNA was extracted using TriFAST™ (VWR, Peqlab, Radnor, PA, USA) according to the manufacturer's suggestions. Isolated RNA was treated with DNaseI (New England Biolabs, Ipswich, Massachusetts) and subsequently purified by phenol: chloroform, chloroform extraction and precipitated with ethanol.

100 ng of DNaseI-treated RNA was rRNA depleted using NEBNext^®^ rRNA Depletion Kit (Human/Mouse/Rat). cDNA libraries were generated with NEBNext^®^ Ultra^™^ II Directional RNA Library Prep Kit for Illumina^®^ (New England Biolabs, Ipswich, Massachusetts) and subsequently sequenced in paired-end mode with 150-bp read length on a NextSeq500 to obtain ∼40 mil. reads (Illumina, San Diego, CA, USA).

### Construction of mislocalized ADAR vectors and immunostaining

Gibson assembly (NEBuider Hifi DNA Assembly Cloning kit, New England Biolabs, Ipswich, MA) and mutagenesis primers were used to generate mutated human flag-ADAR mammalian expression vectors. In short, nuclear ADAR1p150 was prepared by deletion of full NES (ID: P55265-1, 125–150: CLSSHFQELSIYQDQEQRILKFLEEL) (21). The nuclear localization of the mutant was additionally supported by inserting a strong SV-40 NLS (PKKKRKVEDP) replacing the deleted NES. ADAR1p150 ZBDα mutant was made by mutating P193 to A. Cytoplasmic ADAR1p110 was prepared by introducing a single mutation at the position R801A – the crucial residue of the ADAR1-NLS (19). Cytoplasmic ADAR2 was produced by deletion of its N-terminal part bearing NLS (ID: P51400-1, 1–72) (34,35) and the cytoplasmic localization was additionally supported by placing the minimal ADAR1p150 NES (CLSSHFQELSIY, 125–136) (22) instead of the deleted NLS (Supplementary Figure S2).

Localization of resulting plasmids was assessed with immunofluorescence staining using an anti-flag antibody (F7425, Sigma Aldrich, Merck, Kenilworth, NJ, USA) in combination with Alexa 546 secondary antibody (Invitrogen, Thermo Fisher Scientific, Waltham, MA) and counterstained with DAPI. Microscopic confocal sections were taken on Confocal Laser Scanning Microscope FV3000 (Olympus, Tokyo, Japan) and images were processed with ImageJ.

### Western blotting

MEF or HEK293 cells were electroporated with the constructs indicated using NEON electroporation. Cells were harvested after 36 h, lysed in SDS sample buffer and sonicated. Lysates were separated on SDS PAGEs, transferred to PVDF membranes. Membranes were blocked in 1× TBS supplemented with 5% dry milk powder. ADAR1 constructs were detected with an anti ADAR1-antiserum generated in rat directed against human ADAR. The immunogen was a 17kDa fragment covering a region downstream of ZBDß. After incubation with the primary antiserum the blots were detected with a peroxidase-conjugated goat anti rat secondary antibody using BioRad Chemoluminescent detection kit. Alternatively, ectopically expressed ADAR constructs were detected with an anti FLAG antibody (Sigma, St Louis, MI), directed against the N-terminus, or an anti-His antibody (Cell-Signaling, Leiden Netherlands).

### Adar1-RIP

HEK cells were seeded at a density of 6.5 × 10^6^ cells per 150mm dish. After 24 h, two dishes were transfected with 38 μg of plasmid DNA using 90 μl of linear polyethyleneimine (PEI) (Polysciences Warrington, PA, USA) and incubated for 24 h. For harvesting, dishes were kept on ice and washed twice with ice-cold 1× PBS. Cells were subjected to either native RIP or cross-linked RIP procedure:

*Native ADAR RIP* was performed as described previously (36). Only one 100mm dish per condition was subjected to the protocol (transfection mixture was scaled down proportionally). Cells were lysed in 1 ml of ice-cold polysomal lysis buffer (PLB) (100 mM KCl, 5 mM MgCl_2_, 10 mM HEPES (pH 7.0), 0.5% NP40, 1 mM DTT, 50 U of RNase inhibitor (New England Biolabs, Ipswich, Massachusetts), protease inhibitor cocktail (Complete Mini, Roche, Merck, Kenilworth, NJ, USA)). Cell suspension was passed 8 times through a 27.5-G needle. The cell lysate was cleared for 15 min, 16 000g at 4°C. The supernatant was precleaned with 40 μl pre-washed (2 × with PLB) Dynabeads® Protein A (Thermo Fisher Scientific, Waltham, Massachusetts) for 1 h at 4°C. The input sample was collected. Anti-FLAG antibody (F7425, Sigma Aldrich, Merck, Kenilworth, NJ, USA) was added to each sample and rotated overnight at 4°C. 30 μl of Dynabeads® Protein A (pre-washed 2× in PLB) were added to each sample and rotated for another hour at 4°C. Beads were washed 3 times with PLB. 10 × DNaseI reaction buffer was added, and samples were treated with 10 μl of DNase I (New England Biolabs, Ipswich, MA) for 15 min at 37°C. RNA was extracted with 1 ml of TRIFAST as described above for general RNA extraction.

### Cross-linked ADAR1 RIP

For formaldehyde cross-linking, 0,1% formaldehyde in 1× PBS was added to cells and incubated for 10 min at room temperature with gentle mixing. Cross-linking was quenched by adding one-tenth of quenching buffer (2.5 M glycine and 25 mM Tris) (37).

Methylene Blue cross-linking was performed by adding 18 ml of 3 μg/ml Methylene Blue in 1× PBS. Cells were kept on ice and subsequently exposed to visible light for 30 minutes (Kaiser Prolite 5000). Ultraviolet cross-link 2× 800 mJ was additionally applied (UVP Crosslinker, Analytic Jena GmbH, Jena, Germany).

Cross-linked cell pellets were kept at -80°C until cell lysis. The IP-protocol was modified from Ricci *et al.* (37). Pellets were lysed in 1.2 ml (per 2 × 150 mm dishes) of lysis buffer (20 mM Tris–HCl, pH 7.5, 15 mM NaCl, 10 mM EDTA, 0.5% NP-40, 0.1% Triton X-100, 0.1% SDS and 0.1% sodium deoxycholate, protease inhibitor cocktail (Complete Mini, Roche, Merck, Kenilworth, NJ, USA) and 160 U of RNase inhibitor (New England Biolabs, Ipswich, MA) on ice for 10 min.

The suspension was sonicated (Sonopuls HD 2070, Bandelin, Berlin, Germany) at 40% amplitude for a total of 60 s, 2 × 30 s on ice with a 20 s break and subsequently treated with 8 μl/sample Turbo DNaseI for 10 min at 37°C (Invitrogen™, Thermo Fisher Scientific, Waltham, MA). NaCl was adjusted to 150 mM, and the lysate was cleared by two centrifugation steps at 15.000 g for 10 minutes at 4°C. The input samples were collected. The lysate was incubated for 2 h at 4°C with 180 μl of anti-flag agarose beads (50% slurry, ANTI-FLAG® M2 Affinity Gel, Merck, Kenilworth, NJ, USA), prewashed 3× with 1 ml isotonic wash buffer (IsoWB) (20 mM Tris–HCl, pH 7.5, 150 mM NaCl and 0.1% NP-40). Next, beads were washed 2× with 1 ml IsoWB + 0.1% SDS and 0.1% sodium deoxycholate, then 2× with 1 ml with IsoWB. Protein-RNA complexes were eluted with 300 μl of PK-7M Urea buffer (200 mM Tris–HCl pH 7.4, 100 mM NaCl, 20 mM EDTA, 2% SDS and 7 M urea) at 25°C for 2 h with 1000 rpm. Proteins were digested with 2mg/ml of preincubated proteinase K for 2 h, at 25°C with 1000 rpm agitation. RNA was extracted with TRIFAST, treated with DNaseI, followed by Phenol-Chloroform extraction. 10 ng of MB + UV-RIP- and corresponding input- RNA was subjected to rRNA-depletion with NEBNext^®^ rRNA Depletion Kit (Human/Mouse/Rat). cDNA libraries were synthesized using NEBNext^®^ Ultra^™^ II Directional RNA Library Prep Kit for Illumina^®^ (Both: New England Biolabs, Ipswich, Massachusetts) and subsequently sequenced in a paired-end mode with 75bp read length on NextSeq500 (Illumina, San Diego, CA, USA) (to obtain ≈15–20 mil. mappable reads per RIP and input sample).

### RT-qPCR for ADAR-RIP evaluation

20 ng of RIP-RNA and input-RNA was spiked in with 25 ng of Drosophila RNA and reverse transcribed using Maxima H MinusTranscriptase (Thermo Fisher Scientific, Waltham, Massachusetts) and random hexamer primers. qRT-PCR was subsequently performed with primers specific for edited genes and fly sequences using Luna Universal qPCR Mix (New England Biolabs, Ipswich, Massachusetts) and Biorad CFX Connect™ Real-Time PCR Detection System (BioRad, Hercules, CA, USA). qPCR results were normalized on fly-spiked in RNA and a relevant input sample.

### ADAR-RIP NGS data analysis

Sequenced reads were aligned to the human reference genome (H.sapiens/GRCh38) using STAR aligner version 2.5.2a (38). As ADAR1 is known to bind to repeats, the multimapping reads were also included in the analysis following (39).Next, the reads overlapping with ENCODE blacklist regions were removed (40). Peaks were called with MACS2 (version 2.2.7.1) (41) (https://github.com/macs3-project/MACS) separately for each replicate using the IP samples as experimental data and the input RNA as control data. MACS2 does not support strand-specific peak-calling. To circumvent this problem the forward and reverse strand of each chromsomes were treated as two different chromosomes during the peak-calling and merged afterwards. Paired reads are viewed as one fragment in MACS2 and thus produce very long peaks which include also regions with no reads like introns (Supplementary Figure S3). To include only regions with reads, the paired-end reads were split into single-end reads which were then used as input for peak-calling with MACS2 using following command:

macs2 callpeak –treatment {ip} –control {input} –format BED –gsize 6199501436 –outdir {outdir} –name {sample and replicate name} –nomodel –keep-dup all –qvalue 0.01 –extsize 38

The peak lists of replicates were combined by only keeping peaks which are at least adjacent to a peak of another replicate resulting in one peak-list per condition (p150, p110, MOCK). Next, for each of the peak-calling variants the MOCK peaks were subtracted from the p110 and p150 peaks using bedtools (version 2.29.2). If a MOCK peak overlaps a p110 or p150 peak by more than 90% the peak was removed completely, otherwise only the overlap is removed. The final peak-lists were further restricted by minimal peak-size of 40 nt. Additionally, only peaks from the reference chromosomes were included in further analysis (chr1-chr22, X, Y;chrM was excluded).

Peak annotation was done with ChIPseeker (version 1.30.3) using the annotation: GRCh38/ Ensemble 96 (42) according to following priority list: *5UTR, 3UTR, Exon, Intron, Downstream, Intergenic*.

To access the true size of binding regions, the reads were aligned to the transcriptome using Kallisto (version 0.48.0) (43) against Ensemble 96/ GRCh38. The aligned reads overlapping with ENCODE blacklist regions were removed (40). Peaks were called on transcriptomic coordinates with MACS2 (version 2.2.7.1) ((41), https://github.com/macs3-project/MACS) separately for each replicate using the IP samples as experimental data and the input RNA as control data using the following command:

macs2 callpeak –treatment {ip} –control {input} –format BAMPE -gsize 289357337 –outdir {outdir} –name {sample and replicate name} –nomodel –keep-dup all –qvalue 0.01

Peaks were called from paired-end reads directly as only exons were used as an input. Combining of replicas and subtraction of MOCK-peaks was done equally to the genomic peaks. All peaks shorter than 40 nt were removed and only peaks originating from reference chromosomes were included (chr1-chr22, X, Y;chrM was excluded). To omit redundancy, subpeaks of other peaks, originating from different transcripts, were removed before peak-size evaluation of final peak lists.

### NGS data processing and A-to-I editing detection: REDItools

Sequenced reads were inspected with FASTQC (http://www.bioinformatics.babraham.ac.uk/projects/fastqc) and cleaned using FASTP to remove adaptors as well as low-quality regions (44). Cleaned reads were mapped onto the mouse reference genome (GRCm38 assembly), using STAR aligner (38) and a list of known splice junctions from Gencode. Uniquely mapped reads in BAM format and a list of known mouse RNA editing sites from REDIportal were then passed to REDItools to profile known RNA variants per sample (4,45). Annotations are based on Gencode through ANNOVAR (46).

For each candidate site, we calculated the significance of the observed editing level between p110 and 150 samples by applying the *t*-test statistics. Since the t-test has been applied multiple times (>3000 editing sites), to identify significant sites maintaining low the proportion of false positives, we calculated a *q*-value per each site, i.e. the adjusted *P*-value through an optimized FDR approach, using the ‘qvalue’ Bioconductor package (47). We plotted the *q* values versus the significant tests and these last values versus the expected number of false positives in order to choose an appropriate *q* value cut-off and limit the false positives (Supplementary Figure S4). From this *q* value threshold of 0.25 seems most appropriate.

### RT-PCR and A-to-I editing evaluation using sanger sequencing

DNaseI-treated RNA was reverse transcribed using M-MuLV reverse transcriptase (New England Biolabs, Ipswich, MA) and random hexamer primers. cDNA fragments spanning selected editing sites in *Azin1, Pum2, Deptor* and *Nrp1* were amplified (see table: primers), gel eluted, and sent for Sanger sequencing (Eurofins, Luxembourg). The chromatograms were evaluated with SnapGene Viewer software.

### Amplicon sequencing and data analysis

MEFs were electroporated with indicated plasmids for 24 hours. After RNA extraction and cDNA, synthesis amplicons were generated with OneTaq or Q5 Hifi DNA polymerase (both: New England Biolabs, Ipswich, Massachusetts) by 2-step PCR. First, cDNA fragments were amplified with gene-specific primers containing a part of the Illumina adaptor sequence. Second, fragments were barcoded by PCR with primers containing the adapter sequence and unique indexes for multiplexing. Amplicons were gel-purified, pooled and sequenced in paired-end mode with 150 bp read-length on NextSeq550 (Illumina, San Diego, CA, USA). Reads were adapter-clipped using Cutadapt (48) and aligned to the mouse genome mm10 with STAR using public server at usegalaxy.org (38,49). A-to-I transitions were then detected and quantified with Pysam (version 0.16.0.1) (50) (http://www.ncbi.nlm.nih.gov/pubmed/19505943) employing REDIportal set of mouse known-editing sites (http://srv00.recas.ba.infn.it/atlas/index.html) (4).

### Structures of RNA

RNA-structure was predicted using RNAfold 2.0 with default parameters via the Vienna RNA website (51,52).

## RESULTS

To determine the target specificity of ADAR1 isoforms, we employed two different approaches: on the one hand, we identified RNAs that can become edited by ADAR1p150, ADAR1p110 and mutated variants of these proteins upon expression in editing-deficient MEF cells. On the other hand, RNAs that interact with transiently expressed ADAR1 isoforms in HEK293 cells were determined using RIP-seq. Jointly, the two approaches give a robust overview of the ADAR1-interactome and editome.

### ADAR1p150 and ADAR1p110 display specific editing patterns

To evaluate ADAR1 isoform-specific editing preferences Flag&His-tagged human ADAR1p150, ADAR1p110, or RFP were electroporated into *Adar* (ADAR1) and *Adarb1* (ADAR2) deficient mouse embryonic fibroblasts (MEFs). Overexpressed ADAR1 isoforms showed their typical intracellular localization and distinct editing patterns (Supplementary Figure S5). ADAR1p150 can also give rise to a shorter version of the protein that initiates at Met296 and largely resembles ADAR1p110 (18,34,53,54). Therefore, we also tested for the existence of ADARp150 versus ADAR1p110 in our transfected isoforms. To do so, we performed western blots with a human-specific ADAR1 antibody or an antibody against the C-terminal His-tag. The His-tag should also be present if the internal Met296 was used for translational initiation. Interestingly, transfection with constructs encoding ADAR1p150 showed very little production of a shorter version upon detection with the C-terminally located His-tag, suggesting that in cells transfected with ADAR1p150 constructs this would also be the predominant isoform (Supplementary Figures S5C, D, E). Total RNA was extracted 24 h after transfection, followed by ribo-minus depletion and library construction. ≈40 mio. paired-end reads were sequenced per replicate. Reads were mapped to mouse genome mm39. To test for expression of the ectopically expressed ADAR versions, reads were also mapped to the human *ADAR1* cDNAs (Supplementary Table S1: ADAR tpkm). In all transfections, human *ADAR1* versions were expressed at comparable levels to histone *H1f5* or *hnRNPC*. RNA-editing at known sites was evaluated in each sample using REDItools, applying basic filtering criteria: editing rate ≥1% and read-coverage ≥5 reads (45,55).

Only 78 A to G mismatches were found in RFP transfected cells (editase-negative control). These positions presumably arising from SNPs were excluded from further analysis. Editing raised remarkably upon transfection with any ADAR1 isoform ranging from 1.121 to 5.395 edited sites (ES) (Supplementary Figure S6A). Noticeably less editing was detected in the 1st replica of ADAR1p150 as rRNA contamination led to low mRNA coverage precluding efficient detection of editing (Supplementary Figure S6B). Still, when combining all samples and by considering all sites that were covered by more than 5 reads in both experimental and negative control, we could identify 9400 of the ∼100 000 editing sites known in mice (3,4). Detected sites were subjected to further filtering to acquire reliable sets of editing sites for each ADAR1-isoform. Finally, only sites edited in ≥ 2 out of 3 replicas and sufficiently covered in both isoform-datasets (ADAR1p150 and ADAR1p110, average ≥ 10 reads from at least two replicas) were subjected to final analysis (Supplementary Figure S6C). Following these filtering criteria, we collected a relable set of 3137edited sites (Supplementary Table S2_tab MEF editome).

**Figure 1.**
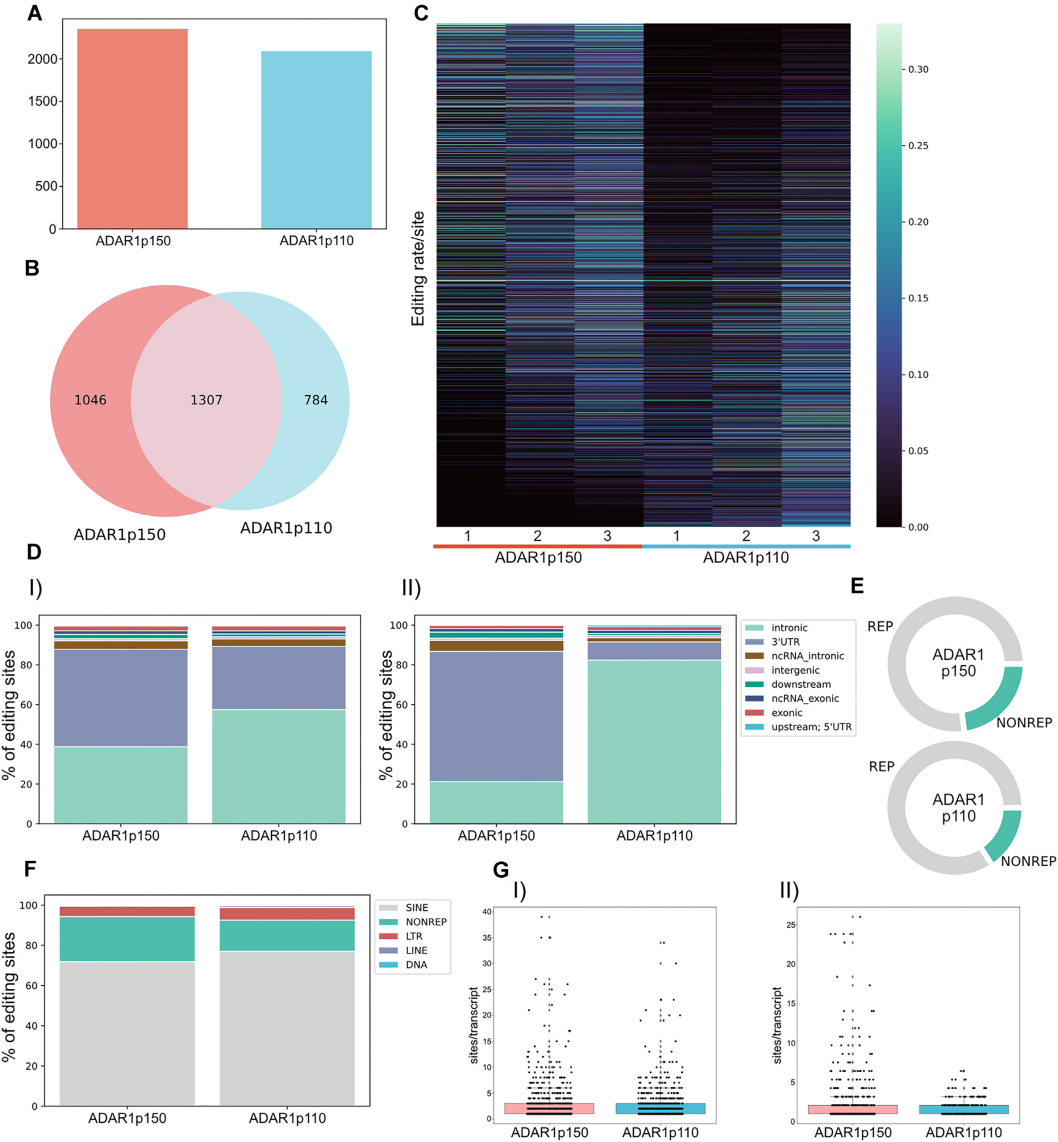
ADAR1p150 and ADAR1p110 display isoform-specific editing patterns. (**A**) 2353 editing sites are efficiently edited (≥1% editing ratio) by ADAR1p150, and 2091 sites are efficiently edited by ADAR1p110. (**B**) Of the detected sites ≈ 42% are efficiently edited by both isoforms (1307); 33% are preferentially edited by ADAR1p150 (1046), and nearly 25% are preferentially edited by ADAR1p110 (784). (**C**) Heat map showing editing ratio of detected editing sites. Despite the large overlap of editing sites between ADAR1-isoforms, the majority of sites still shows a clear preference for one or another isoform. The sites were sorted based on log2-fold difference in editing ratio (p110/p150 in ascending order, showing the sites preferentially edited by ADAR1p150 on the top and sites edited by ADAR1p110 on the bottom of the heatmap. (**D**) Gene-region distribution of editing sites for ADAR1-isoforms. (I) Distribution of all efficiently edited sites. ADAR1p110 edits prevalently in introns. Apart from the intronic regions, ADAR1p150 efficiently edits a prominent portion of 3'UTRs. (II) Distribution of editing sites that are preferentially edited by one isoform over the other (sites with log2FD ≥ 1 for ADAR1p110 and sites with log2FD ≤ –1 for ADAR1p150). Editing sites edited by ADAR1p110 are almost exclusively intronic, whereas a major portion of ADAR1p150 specific sites locates to 3'UTRs. (**E**) ADAR1p150 edits more nonrepetitive regions (∼23%) than ADAR1p110 (∼16%). (**F**) The majority of editing is located within SINE elements. **G**) ADAR1p150 edits more hyperedited regions. I) Boxplot of all detected sites; II) Boxplot of sites preferentially edited by one isoform (sites with log_2_FD ≥ 1 for ADAR1p110 and sites with log_2_FD ≤ –1 for ADAR1p150). Box depicts range from the 25th to 75th percentile. Whiskers depict 1.5× interquartile range from top/bottom of box. Black dots mark number of editing events per transcript. Grey dots depict outliers.

To determine ADAR1 isoform preferences for the assigned sites we tested whether they were edited at ratios ≥1% in at least two of the three replicas and covered by ≥ 10 reads for either enzyme. This revealed a similar number of sites to be edited by either ADAR1 isoform. ADAR1p150 edited 2353 sites while ADAR1p110 could edit 2091 sites at frequencies ≥1% (Figure 1A, Supplementary Table S2; *tab p150 editome*, *tab p110 editome*). 33% of editing sites were preferentially edited by ADAR1p150 and 25% showed preferential editing by ADAR1p110 (preferential editing being defined as ≥1% editing by one isoform and ≤1% editing by the other isoform) (Figure 1B). ∼20% of all sites were exclusively edited by one or the other ADAR1-isoform (ADAR1p150: 335 and ADAR1p110: 267 ES) (Supplementary Figure S7).

To investigate specificity of ADAR1-isoforms in more detail, a fold-difference (log_2_FD) per site was calculated using the average editing level per group (p150 or p110) multiplied by 100 and adding a pseudocount of 1 to avoid an infinite value, potentially caused by a division through zero (Supplementary Table S2). A heat-map depicting sorted values of the log2FD shows that despite the large overlap between ADAR1p150 and ADAR1p110 editing sites, many sites show a clear editing preference for one of the two isoforms (Figure 1 C). To apply even more stringent criteria we calculated a q-value per each site, i.e. the adjusted p-value through an optimized FDR approach, using the ‘*q*-value’ Bioconductor package (47). To do so, *q*-values were plotted against the significance tests and these values versus the expected number of false positives in order to choose an appropriate *q* value cut-off and limit the false positives (Supplementary Figure S8). Supplementary Table S2 includes the *t*-test *P*-value and *q* value per site.

Not surprisingly, nuclear located ADAR1p110 is responsible for the vast majority of intronic editing events. Sites edited by ADAR1p150 show a more complex pattern. Interestingly, a large number of sites targeted by ADAR1p150 are also located in intronic regions. This might be explained by the shuttling ability of ADAR1p150 or by redundant genomic annotations of intronic and exonic regions (56). ADAR1p150 is also editing 3’UTRs with higher efficiency than ADAR1p110, consistent with its predominant cytoplasmic localization (Figure 1D, I). Preferences for intronic regions and 3’ UTRs became even more prominent when only sites with a clear preference for editing by one of the two isoforms (sites with log2FD ≥ 1 for ADAR1p110 and sites with log_2_FD ≤ –1 for ADAR1p150) were considered. There, ADAR1p110 edited almost exclusively intronic sites whereas 3’UTRs became a prominent target for ADAR1p150-editing (Figure 1 D, II).

ADAR1p150 is seemingly more active in nonrepetitive sequences (≈23%) than ADAR1p110 (≈16%) (Figure 1E). Still, the vast majority of editing in repetitive regions by both ADAR1 isoforms occurs in short interspersed nuclear elements (SINEs) and to a lesser extent in LTR and long interspersed nuclear elements (LINEs) (Figure 1F). In ‘hyperedited’ regions that are enriched in repetitive sequences and consequently contain many adjacent potential editing sites ADAR1p150 is considerably more active than ADAR1p110. This is even more evident when only sites with log_2_FC ≥ 1 for ADAR1p110 and sites with log2FC ≤ –1 for ADAR1p150 were considered (Figure 1G, II). Most mRNA molecules spend the majority of their lifespan in the cytoplasm (57). Thus, ADAR1p150, which is mainly cytoplasmic, might have longer access to hyperedited regions (primarily located in 3' UTRs) than nuclear ADAR1p110.

In summary, our data show that ADAR1p110 edits mainly intronic repetitive regions, while ADAR1p150 edits 3’UTRs and hyperedited regions. ADAR1p150 is also more involved in editing of non-repetitive and thus potentially more conserved editing sites.

### ADAR isoform-specificity is partially driven by its cellular localization

**Figure 2.**
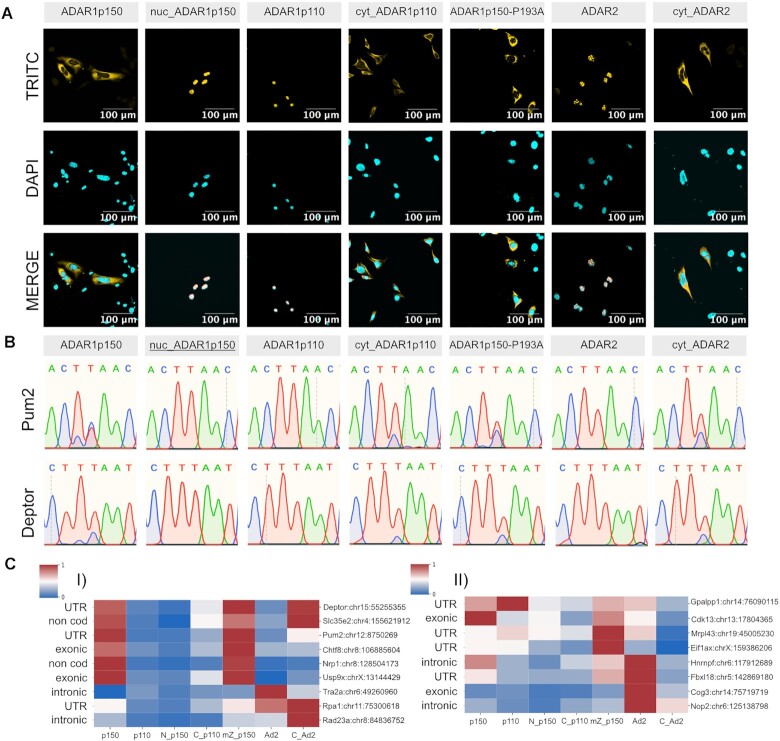
ADAR1-isoform specificity is partially driven by its cellular localization. (**A**) Confocal microscopy images confirm the cellular localization of mislocalized ADAR mutants. A P193A mutation in ZBDα remains cytoplasmic. TRITC channel shows transfected constructs in confocal sections. DNA is stained with DAPI (scale bar: 100 μm). (**B**) Sanger sequencing traces showing the impact of mislocalization and ZBDα mutation on editing-specificity of ADAR1. Pum2 (chr12:8750269) and Deptor (chr15:55255355) are efficiently edited by wild-type ADAR1p150 but not by wild-type ADAR1p110. Cytoplasmic ADAR1p110 and cytoplasmic ADAR2 also edit both selected targets, however, only at one of the two adjacent sites. In contrast, nuclear ADAR1p150 does not show any editing of selected targets. The P193A mutation does not affect Deptor editing but leads to reduced editing at one of the two sites in Pum2. The reverse strand is sequenced showing T to C conversion in the chromatogram. (**C**) Heat maps of editing by wild-type and mutated ADAR versions at selected editing sites detected by amplicon-seq. I) Impact of mislocalization on editing of ADAR1p150 targets. II) Impact of mislocalization on editing of ADAR1p110 targets. At ADAR1p150 sites, the P193A mutation does not affect editing but nuclear localization of ADAR1p150 reduces editing. At the same time, cytoplasmic localization of ADAR1p110 or ADAR2 allows editing of ADAR1p150 sites (p150 = ADAR1p150, p110 = ADAR1p110, N_p150 = nuclear ADAR1p150, C_p110 = cytoplasmic ADAR1_p110, mZ_p150 = mutant ZBD in ADAR1p150, Ad2 = ADAR2, C_Ad2 = cytoplasmic ADAR2). Names and chromosomal positions of editing sites are indicated at the right of the heat map.

Having identified distinct editing patterns of ADAR1-isoforms, we aimed to determine the characteristics of ADAR1 isoforms that drive substrate specificities. ADAR1p150 differs from ADAR1p110 by an extended N-terminal region unique to p150. The p150-specific N-terminus harbors a nuclear export signal (NES) and a Z-DNA binding domain (ZBDα) that can interact with double-stranded nucleic acids in Z-conformation. To test whether cytoplasmic localization or the presence of ZBDα are driving ADAR1p150 specificity, we constructed mutant versions of both ADAR1 isoforms. Specifically, we generated nuclear ADAR1p150, cytoplasmic ADAR1p110 and ADAR1p150 bearing a P193A mutation in ZBDα. The P193A mutation affects ZBDα function (58). We also constructed a cytoplasmic version of ADAR2 (Supplementary Figure S2). Cellular localization of all mutants was confirmed by immunofluorescent staining (Figure 2A) and western blotting (Supplementary Figure S9). Next, we studied the impact of mislocalization on ADAR editing specificity. Using Sanger sequencing, we evaluated editing in *Pum2* and *Deptor* upon transfection of different ADAR versions into MEFs (ADAR1^−/−^;ADAR2^−/−^). In our initial analysis *Pum2* and *Deptor* were edited at two adjacent positions by wild-type ADAR1p150 but not by wild-type ADAR1p110 and wild-type ADAR2. However, nuclear ADAR1p150 did not edit any of the selected sites, whereas cytoplasmic ADAR1p110 and even cytoplasmic ADAR2 did edit these substrates on one of the two adjacent positions. The ZBDα P193A mutation did not affect editing at both studied substrates, but led to reduced editing at one of the two adjacent nucleotides when compared to wild-type ADAR1p150 (Figure 2B). This finding suggested that the specificity of ADAR1p150 isoform is prevalently driven by its cellular localization and, thus, might be substituted by any cytoplasmic editase.

### Amplicon-seq of wild-type, mislocalized and mutated ADAR variants

To further assess the effect of mislocalized and mutated ADARs on their editing preferences, amplicon-seq was performed for a set of substrates. To do so, ADAR1^−/−^; ADAR2^−/−^ MEFs were electroporated with plasmids expressing mutated and wild-type ADAR versions. An RFP expressing vector was used as a negative control. 17 editing targets were selected based on their preferential editing by ADAR isoforms and their comparable read coverage. Further, we aimed at picking editing sites located in exons, introns, 3’UTR, repeats and nonrepetitive regions. In doing so we picked 9 targets that were preferentially edited by ADAR1p150 and 8 targets by ADAR1p110 (Supplementary Table S3). However, it should be noted that sites preferentially edited by ADAR1p110 are strongly enriched for intronic sequences. Obviously, nuclear retained intronic sequences would not have a chance to be edited by cytoplasmic ADAR versions. Therefore, by including exonic ADAR1p110 substrates we also included sites with a relatively low preference score (see Supplementary Table S3). Experiments were performed in triplicate using 5 μg of plasmid DNA for electroporation. A fourth replicate was was electorporated with 10 μg of plasmid to control for effects caused by different expression levels. After STAR alignment, background signals occurring in the negative control were subtracted, and the remaining sites were subjected to further filtering. Sites edited ≥0.5% by any mutant protein were collected into the final dataset. Overall editing in cells electroporated with 5 μg DNA was low. However, editing patterns remained similar as for cells transfected with 10 μg of DNA (Supplementary Figures S10, S11). To compensate for the heterogeneity of editing levels by different constructs, editing levels were normalized. Maximum editing rates of a particular site achieved by any of the constructs used were set to one (1) and the editing values for remaining editases was proportionally adjusted (Figure 2C; Supplementary Figure S12; Supplementary Table S4). These analyses showed that many ADAR1p150 targets are also editable by cytoplasmic ADAR1p110 and cytoplasmic ADAR2 (*Pum2*, *Deptor* and *Slc35e2*) (Figure 2B, C; Supplementary Figure S12). This clearly indicates that a fraction of sites edited by ADAR1p150 can also be edited by other cytoplasmic ADARs. The P193A mutation did not affect the ADAR1p150 editing pattern. Only the site in *Nrp1* and to a minor extent the site in *Chtf8* were primarily edited by wild-type ADAR1p150 and by ADAR1p150 carrying the ZBD*α* mutation but not by other cytoplasmic editors. The site in *Nrp1* was also validated by Sanger sequencing (Supplementary Figure S13). Thus, *Nrp1* and *Chtf8* appear to be true ADAR1p150 editing substrates that specifically require ADAR1p150 for editing that cannot be substituted by other cytoplasmic editors. Individual sites in *Tra2a*, *Rpa1* and *Rad23* were preferentially edited by ADAR2 and cytoplasmic ADAR2. The initial experiments did not include ADAR2, and hence co-occurrence ADAR2 and ADAR1p150 editing-sites cannot be excluded. Nonetheless, the editing sites in *Rpa1* and *Rad23a* showed higher editing rates for ADAR1p150 than for ADAR1p110 (Figure 2C).

The situation was less clear for targets that displayed a preference for editing by ADAR1p110. *Gpalpp1*, *Mrpl43* and *Eif1ax* are all sites in UTRs that also were preferentially edited by ADAR1p110 in amplicon seq. However, they were also well edited by ADAR1p150 carrying a mutant in the ZBD and to a lesser extent by ADAR2. The intronic sites in *Hnrnpf* and *Nop2* showed a clear preference for ADAR2 consistent with their nuclear localization, suggesting that their original occurrence in the ADAR1p110 dataset was probably an artefact. The site in *Cog3* shows a slight preference for ADAR1p110 over ADAR1p150 but is in fact a protein recoding event that is preferentially edited by ADAR2. *Cdk13* sites appeared preferentially edited by cytoplasmic ADAR1p150, the ZBD mutant but also by ADAR2. The editing site in the UTR of *Fbxl18* showed a clear preference for ADAR2 while all other enzyme variants show only very low editing rates on this substrate. Overall, amplicon seq indicates that while substrates for ADAR1p150 were quite accurately picked by our approach, substrates for ADAR1p110 are of low specificity and have a high false discovery rate. Moreover, substrates of ADAR1p150 can also be edited by other cytoplasmic ADARs, while some substrates originally identified as ADAR1p110 substrates seemingly are substrates of ADAR2 or can be edited rather promiscuitively (Figure 2C, Supplementary Figure S12).

### RIP-seq identifies a distinct binding distribution of ADAR1-isoforms

After having identified the editing map of ADAR1 isoforms in the mouse, we wanted to identify RNAs interacting with ADAR1 isoforms in humans. As ADARs bind to structured regions in RNAs that are frequently formed between complementary regions within 1 kb of each other, we aimed at capturing these extended ADAR-interacting regions (59). To do so we determined the binding regions of ADAR1 isoforms to human substrate RNAs in HEK293 cells with RIP-seq using ectopically expressed human FLAG-ADAR1 isoforms. Expression was monitored by immunofluorescence (Supplementary Figure S14). To determine optimal RIP-seq conditions, we compared the original RIP protocol (60) with crosslink-RIP using either formaldehyde (fRIP) or methylene blue photo-crosslinking in combination with UV (MB + UV) (Supplementary Figure S15) (37,61,62). All examined protocols were optimized using FLAG-ADAR1p150.The efficiency of the used protocols was determined by quantifying the fold-enrichment of known ADAR1-substrates in IP-fractions versus corresponding inputs by qPCR of the exonic editing site (ES) in *Azin1* (chr8:103841636), a non-repetitive ES in the 3’UTR of *Pum2* (chr2: 20450819), and the heavily edited 3’UTR of *Nicn1*. We observed a 10–20-fold enrichment of all 3 test substrates in MB + UV RIP while the other tested conditions only showed moderate, 2–3-fold enrichment (Supplementary Figure S15). Thus, MB + UV seems a useful cross-linking method to detect ADAR1-RNA interactions.

Next, MB-UV RIP-seq was performed for human FLAG-ADAR1p150 and FLAG-ADAR1p110 isoforms in triplicates with a duplicate set of MOCK- transfected cells as a control. Specific target enrichment in IP-fractions was evaluated in comparison to the relevant input (Supplementary Figure S16). ADAR1p110-IP, when compared to ADAR1p150-IP, displayed only mild enrichment of selected targets. This indicates that the examined targets are prevalently bound and likely edited by ADAR1p150. Editing was indeed confirmed for *Azin1* (chr8:103841636) by Sanger sequencing (Supplementary Figure S16).

**Figure 3.**
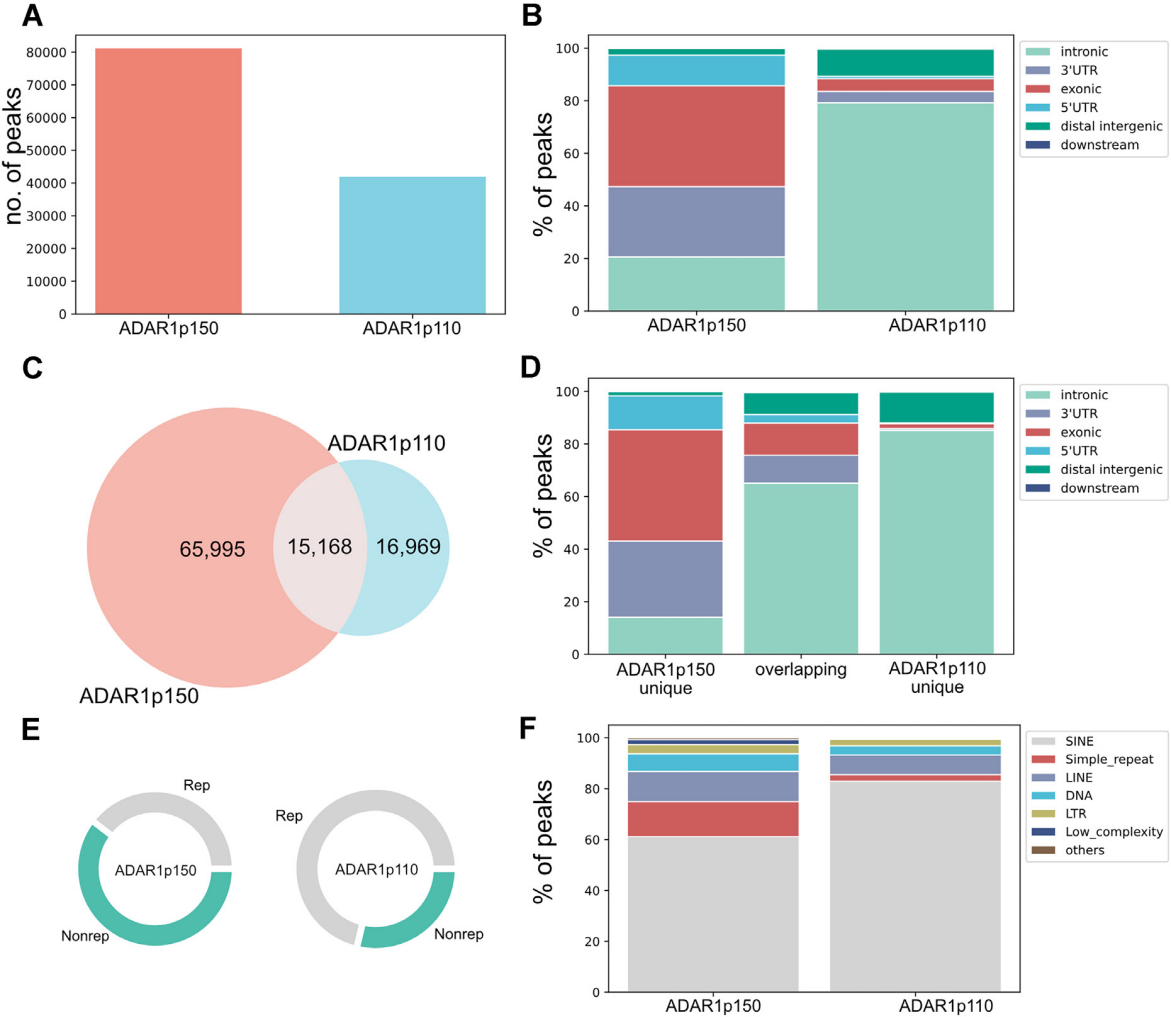
RIP-seq identifies distinct binding regions for ADAR1-isoforms. (**A**) Number of peaks identified by peak-caller after passing filtering criteria. ADAR1p150: 81.163, ADAR1p110: 41.900. (**B**) Peak-distribution in gene regions. ADAR1p110 mainly binds in introns whereas ADAR1p150 shows more complex binding involving exons and 3'UTRs. (**C**) Isoform-specific and overlapping peaks (depicted ADAR1p150 overlap – 15.168 overlapping peaks). (**D**) Distribution of isoform-specific and overlapping peaks to gene regions. (**E**) Peak distribution to repeats and non-repetitive regions. While only 29% of ADAR1p110-peaks cover unique regions >60% of ADAR1p150-peaks span non-repetitive regions. (**F**) The majority of interacting repeats locates to SINE elements, especially in the case of ADAR1p110. ADAR1p150 binds more diverse repeats, including also simple repeats, LINE and DNA transposons.

IP- and relevant input-samples were used for ribo-minus NGS library construction. 15−20 mio paired-end reads were sequenced per sample. To identify ADAR1-isoform binding substrates and regions peaks were called using MACS2 (41). Peaks are here defined as regions where the normalized read-count of the IP-sample is enriched in comparison to the normalized read count of the respective input-sample. Peaks that also appeared in the MOCK control were subtracted from ADAR1p150 and ADAR1p110 peaks to exclude unspecific peaks. This resulted in 81.163 peaks for ADAR1p150 and 41.900 peaks for ADAR1p110 (Figure 3A, Figure 1 - Supplementary Table S5).

Peak-size distribution varied remarkably between isoforms. The average of ADAR1p150 peaks was 951 nts while the average of ADAR1p110 peaks was 328 nts (Figure 3; Supplementary Figure S17 A). The peak-size was computed on genomic coordinates where peaks are affected by splicing. To access the true size of binding regions, we aligned the reads to the transcriptome and performed the peak-calling using transcriptomic coordinates. This resulted in approximately the same peak-size distribution for both isoforms: the average size of ADAR1p150 peaks is 885 nt and the average size of ADAR1p110 peaks is 855 nt supporting our presumption that MACS2 peak-caller is a suitable tool to identify peaks in the ADAR1 RIP-seq experiment (Supplementary Figure S17B; Supplementary Table S6). As expected, transcriptomic peaks appeared longer since they are not split by splicing like the peaks called on genomic coordinates.

The considerable discrepancy in the identified peak numbers: 81.163 ADAR1p150-peaks versus 41.900 ADAR1p110-peaks can likely be explained by the higher reaction dynamics of ADAR1p110 binding which mostly occurs co-transcriptionally in the nucleus (7,63). Thus, nuclear binding of RNAs by ADAR1p110 is more transient resulting in less precipitated RNAs than RNAs bound by cytoplasmic ADAR1p150 which likely will have a longer interaction time. Indeed, the majority of ADAR1p110 peaks is located in introns which corresponds well with its predominantly nuclear localization. In contrast, ADAR1p150 peaks largely fall into exonic regions, including a large fraction of 3’ UTRs (Figure 3B). The ADAR1p150- and ADAR1p110 peak-set showed a prominent overlap of 15.168 peaks for ADAR1p150, 24.931 peaks for ADAR1p110, respectively (ADAR1p150 peaks overlap with more than 1 ADAR1p110 peak) (Figure 3C). Thus nearly 60% of all ADAR1p110 peaks overlap with almost 19% of all ADAR1p150 peaks. This huge overlap reflects the promiscuous character of editing mediated by ADAR1. This finding also supports our editome data from mouse cells, where the majority of the detected sites were edited by both ADAR1-isoforms, albeit, with different editing rates that mostly showed a preference to a particular ADAR1-isoform. Distribution of the binding regions differed even more dramatically upon excluding overlapping peaks. Regions uniquely bound by ADAR1p110 showed nearly exclusive intronic distribution, whilst exonic and 3’UTR regions became even more prominent when peaks uniquely bound by ADAR1p150 were analyzed (Figure 3D).

More than 60% of ADAR1p150-peaks span non-repetitive regions, whereas only 29% of ADAR1p110-peaks lie in non-repetitive regions (Figure 3E). This observation greatly correlates with the gene-region distribution of the peaks. Whilst ADAR1p110 binds mostly introns that contain a high portion of repeats, ADAR1p150 also binds in 3’UTRs and exons. The majority of the interacting repeats accounts for SINE elements in the case of both isoforms. However, ADAR1p150-peaks span also a significant portion of simple repeats, LINE and DNA transposons (Figure 3F). In summary, RIP seq experiments identified distinct binding regions for ADAR1-isoforms. While ADAR1p110 binds mostly intronic regions, ADAR1p150 also binds to exons and 3’UTRs.

### Mouse edited substrates and human MB-UV RIP-seq detect common substrates

Next, we aimed to determine a possible overlap between mouse substrates edited by ADAR1 isoforms and human sequences bound by ADAR1 isoforms. When looking at gene sets we found an overlap of 730 genes for ADAR1p150 and 559 genes for ADAR1p110. This difference could be caused by the lower number of peaks detected by ADAR1p110 RIP-seq.

**Figure 4.**
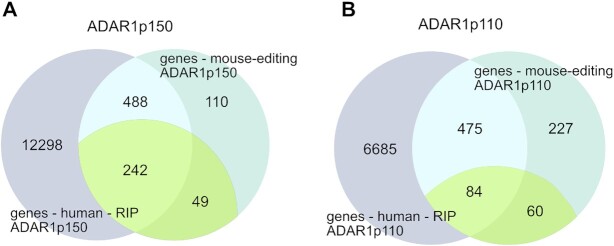
Intersection of regions bound by ADAR1 isoforms identified by ADAR1 RIP-seq in human cells with editing regions identified in mouse genes. (**A**) More than 80% (242) of the genes containing non-repetitive editing sites were detected in both experiments performed with ADAR1p150. (**B**) Only ∼59% (84) was identified between two experimental approaches for ADAR1p110. In purple: genes that were identified by RIP-seq in human cells; turquoise: genes that were identified as editing targets in MEFs; yellow-green: genes containing editing sites in non-repetitive regions and those that were edited in MEFs.

Editing is very abundant in human and occurs in nearly all genes due to the high repeat content of the human transcriptome (2). While editing is also high in mouse repetitive regions, the repeat repertoire between mouse and human is very different due to different repeat types and genomic insertion (59). Thus, we next focused on genes that were edited in non-repetitive regions. This resulted in 291 genes edited by ADAR1p150 and 144 genes edited by ADAR1p110. This way, we expected to find ADAR1 isoform-specific editing targets that are conserved between mouse and human. Indeed, we could identify 84 overlapping genes edited or bound by ADAR1p110. Strikingly, more than 80% (242) of the non-repetitive ADAR1p150 targets were common between human RIP-seq and mouse editing detection experiments (Figure 4, Supplementary Table S7). These results are in agreement with our previous observation that ADAR1p150 strongly edits 3’UTRs that are generally more conserved than introns which are predominantly edited by ADAR1p110.

## DISCUSSION

In this study, we identify ADAR1-isoform-specific editing- and binding characteristics. We identify RNAs that are selectively edited or bound by either ADAR1 isoform. We show further that a large fraction of RNAs can be edited and bound by either isoform. Our study revealed further, that cellular localization is a major factor driving isoform-specific editing patterns. Still, editing of isoform-specific substrates is not exclusively governed by the enzyme's localization, indicating that also other factors likely RNA-binding or competing RNA-binding proteins mediate ADAR1 substrate-specificity.

Upon restoring ADAR1-isoform expression in editing-deficient MEFs 9.400 editing sites of the >100 000 known editing sites in the mouse (available on REDIportal (http://srv00.recas.ba.infn.it/atlas/search.html) could be detected (4). After comprehensive filtering, a high-quality dataset of more than 3000 editing sites was obtained. Of these ∼2000 were efficiently edited by one or another ADAR1-isoform. While ADAR1p110 shows a strong preference for intronic sites, most ADAR1p150 sites are located in 3’UTRs. As expected, the majority of editing sites is located in SINE elements. Nevertheless, ADAR1p150 showed a bigger portion of editing also in non-repetitive regions, which suggests that ADAR1p150-sites might be more conserved among species. Furthermore, ADAR1p150 is also more active in hyperedited regions, likely due to the spatio-temporal advantage of its cytoplasmic localization. We also observed significant levels of intronic editing events by ADAR1p150. This may largely be attributed to the nucleo-cytoplasmic shuttling ability of ADAR 1p150 (19,21,56).

We also identified ADAR1p110-specific editing sites. Still, we cannot exclude that these sites might also be edited by ADAR1p150 as sequencing depth is generally lower in intronic regions that are preferentially edited by ADAR1p110. Indeed, the editing sites in *Gpalpp1* (chr14:76090115) that were identified as exclusive ADAR1p110 sites were later shown to be also edited by ADAR1p150 using amplicon-seq with higher coverage (Figure 2C; Supplementary Table S4). A similar observation was made by Sun et al. when studying editing in HEK cells. Here, several putative ADAR1p110 sites also turned out to be targeted by ADAR1p150 when sequencing depth was increased (54). Moreover, overexpression of one ADAR1 isoform in the absence of another isoform may lead to an overestimate of sites edited by a specific isoform. However, expression of the transfected constructs was moderate (Supplementary Table S1) giving rise to average editing levels of 7% for p110 and 8% for p150 at the investigated target sites. Thus, while overexpression may lead to a distortion of the exact percentage of editing. The preference of editing sites for one or the other isoform is likely not affected by the ectopic expression used here. Another point that cannot be excluded is the inadvertent production of ADAR1p110 from our ADAR1p150 constructs by translation initiation from Met296 as reported by Sun et al.(54). Indeed, an antibody directed against human ADAR1 detects two bands in ADAR1p150 transfected cells. However, as western blots of the same lysates probed with an antibody against a C-terminal His tag show mainly production of ADAR1p150 and very little ADAR1p110, it seems as if only minor amounts of ADAR1p110 were produced (Supplementary Figure S5C–E). Moreover, even if the ADAR1p150 editome had spill-over from the ADAR1p110 editome, this would lead to reduction of the ADAR1p150 specific target set. Still, even a smaller ADAR1p150 target set would still be specific for this ADAR1 isoform.

Lastly, our study relied on a high-confidence set of editing sites previously established in the mouse (3). This dataset only contains sites that are edited with a minimal frequency of ∼10%. However, editing may also occur at low levels at stochastic sites and the impact of these sites on Mda5 activation goes largely unnoticed to this point. Thus, our approach may miss such lowly edited sites. Detection of low abundant editing sites using recently published approaches may therefore reveal additional targets that are specific for ADAR1p150 or ADAR1p110 (64).

Still, the driver of ADAR1p150-specificity remained elusive. The unique presence of a ZBD and a predominant cytoplasmic localization are two exclusive features of ADAR1p150. ZBDα recognizes the unusual Z-conformation in RNA or DNA and might contribute to ADAR1p150 selectivity (65–67). A P193A mutation in human ADAR1 was connected to AGS (58) and studies in mice confirmed that mutations in ZBDα lead to MDA5 activation and contribute to editing selectivity of ADAR1p150 (25–28). A mutation of ZBDα mainly affects editing in SINEs (25) that are regions of putative Z-RNA conformation (68). Surprisingly, a P193A mutation of ZBDα in ADAR1p150 tested here only marginally affected editing of the selected substrates. A recent study by Maurano et al. showed that P195A, mimicking human P193A is haploinsufficient (28). Similarly, mice bearing homozygous N175A/Y179A which considerably disturbs Z-RNA binding (69,70), are viable but trigger an MDA5/MAVS-related IFN-response and show reduced editing of SINEs (25,27). In conclusion, ZBDα probably facilitates editing of certain substrates. The fact that we only observe minor changes in the editing behavior of P193A mutants might therefore be explained by the limited number of substrates investigated by us, thereby possibly missing substrates that crucially depend on the presence of P193A as described (25).

Nuclear editing occurs co-transcriptionally and is therefore transient (7,63). Cytoplasmic editing by ADAR1p150 might therefore be more extensive also targeting hyperedited regions. Interestingly, we observed that many ADAR1p150-specific sites are edited by mutant versions of ADAR1p110 and ADAR2 that localize to the cytoplasm. This finding suggests that the specificity of ADAR1p150 is at least partly driven by its cytoplasmic localization. However, a few sites, especially sites in *Nrp1*, seem insensitive to cytoplasmic versions of other editing enzymes and require exclusive ADAR1p150-editing. *Nrp1* is a cell surface glycoprotein that serves as a receptor for VEGF. Recent studies show that *Nrp1* serves as a SARS-CoV-2 coreceptor facilitating virus cell entry and infectivity (71–73). The role of editing in the 3’UTR of *Nrp1* has not been studied even though the editing sites and surrounding region share surprisingly high conservation amongst mammals (Supplementary Figure S18). While editing of the 3’UTR (chr10: 128504171, 128504172, 128504173) in mouse is annotated in REDIportal, the corresponding human sites are not included in REDIportal or DARNED. Nonetheless, the corresponding human site for ch10:128504173−chr10:33467752 (hg19) was identified as an exclusive ADAR1p150 target in HEKs by Sun *et al.* (54). This indicates that the *Nrp1* 3’UTR carries conserved editing sites that are edited by ADAR1p150. Apart from *Nrp1*, an examined site in *Chtf8* seems to be also insensitive to nonspecific cytoplasmic editing (Figure 2).

Using a modified RNA-IP (RIP)-seq protocol that involved methylene blue and UV cross-linking, we could improve the pull-down efficiency of ADAR-associated sequences. Using this technique, we found distinct binding regions for ADAR1-isoforms. While ADAR1p110 shows strongly enriched binding to introns, ADAR1p150 did efficiently capture exons and 3’UTRs. To compare our RIP-Seq results obtained from HEK293 cells with a recently published ADAR1-isoform-specific editome generated in HEK293 cells (54) we employed CrossMap (v0.5.4) (74) to convert the set of identified editing sites to the GRCh38 assembly. Next, we defined testing groups: I) sites edited exclusively by ADAR1p150 (in at least 2 out of 3 replicas)- which yielded 2806 sites and II) sites edited by ADAR1p150 (in 2 out of 3 replicas) and ADAR1p110 (at least in 1 replica), which yielded in 4939 sites (Supplementary Table S8). Sun et al also identified a limited number of sites edited by ADAR1p110. However, since the editing specificity at these sites was not further confirmed we omitted them from further evaluation.

**Figure 5.**
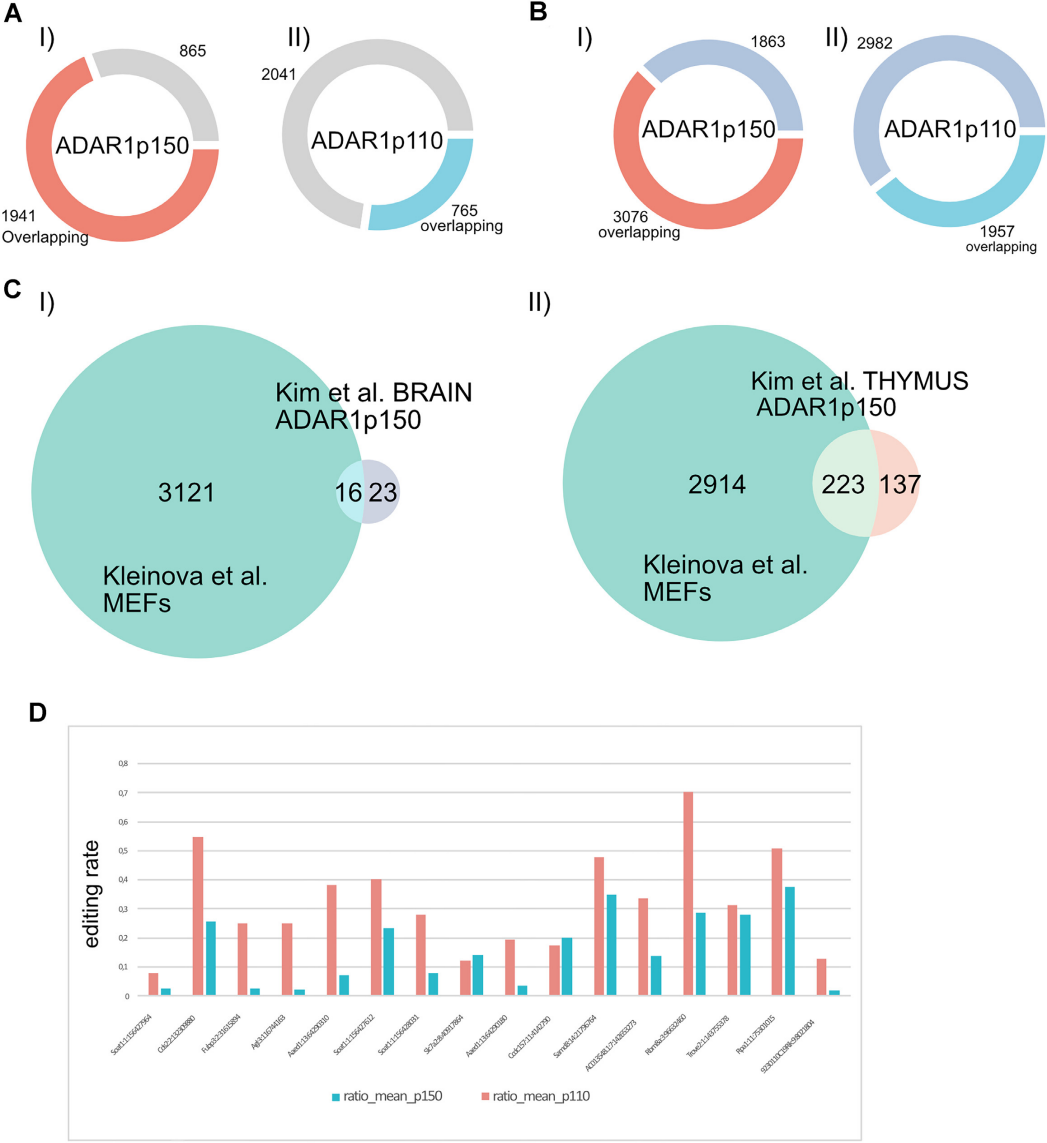
Discussion supporting figure. (**A**, **B**) Intersection between the peaks identified in this study by MB-UV RIP-seq in HEK cells and editing sites identified by Sun *et al.* in HEK cells (54). Red and light-blue sections of doughnut plots display the portion of editing sites overlapping with RIP-seq peaks. (A, I) Overlap of ADAR1p150-specific editing sites and ADAR1p150-peaks. (II) Overlap of ADAR1p150-specific editing sites and ADAR1p110-peaks. (B, I) Overlap of editing sites targeted by both ADAR1 isoforms and ADAR1p150-peaks. (II) Overlap of sites edited by both ADAR1 isoforms and ADAR1p110-peaks. (**C**) Comparison of ADAR1-editome identified in MEFs and ADAR1p150-specific editing sites detected in the brain and the thymus of ADAR1p110^−/−^ ADAR2^−/−^. (I) Editing sites identified as ADAR1p150-specific in the brain. (II) Editing sites identified as ADAR1p150-specific in the thymus. (**D**) ADAR1p150 and ADAR1p110-mediated editing ratios detected in MEFs for sites that overlap with ADAR1p150 sites in the brain identified by (Kim *et al.*, 2021).

Next, using bedtools-intersect (v2.30.0), we identified editing sites that directly overlap with RIP-seq peaks identified by us (75). Indeed, we found a massive overlap of ∼70% between sequence peaks defined by ADAR1p150 binding and editing sites that were selectively edited by ADAR1p150 as detected in the Sun et al., study (54) (Figure 5A). We also found an overlap of ∼62% between sequences bound by ADAR1p150 and editing sites targeted by both ADAR1p150 and ADAR1p110. Consistently, the overlap between ADAR1p110-peaks and both examined editing site sets was considerable smaller: ∼27% with ADAR1p150 editing sites and ∼ 40% with the combined ADAR1p150 & ADAR1p110 editing site set (Figure 5B) (Supplementary Table S8). Overall, this comparison shows that ADAR1p150 RIP-seq peaks have a larger overlap with sites edited by ADAR1p150 while there seems less overlap with sites precipitated by ADAR1p110 and those that are edited by ADAR1p150 and ADAR1p110. However, it should also be noted that RIP-seq identified significantly fewer peaks for ADAR1p110. This observation, together with preferential intronic binding of ADARp110 versus ADAR1p150 binding to exonic 3’UTRs indicates that the identified peaks provide a valuable dataset of high specificity. Still, we cannot exclude that overexpression of ADAR1p110 leads to promiscuous binding and competition for substrates that are otherwise exclusive for ADAR1p150. Apart from the N-terminus of ADAR1p150, ADAR1-isoforms are identical (9,53). Therefore, an overlapping affinity for the same substrates can be expected. RIP-seq of endogenous ADAR1 might improve the substrate-specificity of the procedure. Still, ADAR1p150 is expressed at a very low level unless stimulated by IFN (9). Also, specific antibodies recognizing only the N-terminus of ADAR1p150 are scarce. Therefore, experimental conditions would require adequate optimization to specifically detect binding sites of ADAR1 isoforms.

ADAR1 deficient mice and loss of function mutations in human ADAR1 share similar phenotypic characteristics, including massive IFN-overproduction (16). In our current study, we employed: (I) ADAR1-isoform-specific reconstitution of ADAR1 editing in mouse MEF cells. And (II) ADAR1-RIP-seq performed in human cells. Thus, to compare the two resulting datasets we focused on genes with editing sites in non-repetitive regions, assuming that such sites may exhibit more genomic conservation between mouse and human. While only 84 such genes overlap between the two datasets for ADAR1p110, almost 250 such genes are common between ADAR1p150 editing analysis and ADAR1p150 RIP-seq. Closer inspection revealed many interesting ADAR1p150 substrates, including a highly-conserved recoding editing site in *Azin1* (chr15:38491612) recently described as ADAR1p150 substrate also by Kim *et al.* (Supplementary Figure S19, Supplementary_Table S2_MEF_editome) (18). The protein recoding editing in *Azin1* is extensively studied for its role in many cancer types and lately also for its involvement in hematopoietic stem cell differentiation (76–79).

Still, it appears unlikely that one individual substrate is responsible for the embryonic lethality observed in ADAR1p150 deficiency as a result of MDA5 activation (80). Instead, editing at multiple sites might cause the indispensability of ADAR1p150-editing. We could clearly observe a correlation between ADAR1-editing in mouse and ADAR1-binding in human cells. While ADAR1p110—editing and binding fall mostly into intronic regions, 3’UTRs seem a common prominent substrate for ADAR1p150 binding and editing. 3’UTRs often contain inverted SINE elements and thus form dsRNA structures that present ideal substrates for ADAR1 with many putative editing sites (2,81). Importantly, such long dsRNA stretches also present ideal triggers for MDA5-activation (82). In our experiments these hyperedited regions were preferentially edited by ADAR1p150. This indeed raises the question of whether sufficient editing of these hyperedited 3’UTRs is a key and essential feature of ADAR1p150-mediated editing. A recent study from Kim et al. showed only limited editing sites that are exclusively edited by ADAR1p150 to prevent MDA5-activation (18). In this study, mouse knockout models including an ADARp110 specific knockout in the presence or absence of an additional ADAR2 deficiency were used to characterize the remaining ADAR1p150-mediated editing in brain and thymus. To evaluate overlap with our MEF-editome, ADAR1p150-editing sites identified by Kim et al. were filtered using stringent read-coverage criteria (read coverage -mean ≥ 10 reads in all genotypes), similar to those used in this study. This resulted in a set of putative ADAR1p150 editing sites in the brain and the thymus (Supplementary Table S9). Next, we intersected the exclusive-ADAR1p150 editing pattern identified in that study with the ADAR1 editome identified here in MEFs. We found a large overlap between the datasets. 16 sites, out of 39 in the brain and 223 out of 360 in the thymus (Figure 5C, Supplementary Table S9). Although the majority of sites originally identified in the brain showed a great preference for ADAR1p150-editing in MEFs, none of them was exclusively edited by ADAR1p150 in our setup (Figure 5D). In the thymus editing set, we found 21 sites exclusive for ADAR1p150 editing and additional 52 sites with a high (–2 ≤ log_2_FD) preference for ADAR1p150-editing in isoform-transfected MEFs (Supplementary Figure S19). The limited number of exclusive ADAR1p150 sites identified by Kim et al. might in part result from a limited sequencing depth and might therefore be increased by deeper sequencing. In fact, the coverage of examined sites was about one third higher in our experiment (average coverage ≈ 78 reads/site -this study- versus 59 reads/site in Kim et al), although the total number of sequenced reads was comparable (Supplementary Figure S20). Most overlapping sites fall into 3’UTRs (Supplementary Figure S21), which might again indicate that those are the essential ADAR1p150 sites that have to be masked by editing to prevent MDA5-triggering under physiological conditions. However, in this study we are also overexpressing ADAR1 isoforms. Therefore, we cannot formally exclude that overexpression of e.g. p110 also leads to editing sites that would normally be preferentially edited by p150 and vice versa. To clarify this point, additional sequence analysis of isoform-specific knock-out tissues will need to be performed.

Taken together, we show that editing specificity of ADAR1p150 is at least partly driven by its cytoplasmic localization. A proper genetic mouse model with a physiological expression of either cytoplasmic ADAR1p110 or cytoplasmic ADAR2 in an otherwise editing-free background would be required to fully estimate the role of the cytoplasmic localization and ZBDα in ADAR1p150-specificity. Additionally, we identified sites with no obvious sensitivity to unspecific cytoplasmic editing; putative exclusive ADAR1p150 sites. Based on our data, we hypothesize that promiscuous cytoplasmic editing occurs mostly in long dsRNA stretches, whereas exclusive ADAR1p150 editing sites lie in structures with more complex folding patterns. Loss of ADAR1 has recently been shown to help to overcome PD1 checkpoint blockade (83). It will thus be interesting to see whether the substrates identified here are involved in overcoming PD1 blockage when expressed in an unedited state.

## DATA AVAILABILITY

NGS data generated in this study are available at GEO with the following accession numbers:

ADAR1 RIP-seq data: GSE188937

Editing data of transfected MEFs: GSE188842

## Supplementary Material

gkad265_Supplemental_Files
