# Supplementary material for: The ADAR1 editome reveals drivers of editing-specificity for ADAR1-isoforms
Source: Nucleic Acids Res. 2023 Apr 7;51(9):4191–207. doi: 10.1093/nar/gkad265 (PMC10201426; doi:10.1093/nar/gkad265)
Supplement: gkad265_Supplemental_Files [file gkad265_supplemental_files.zip › Kleinova et al., Supplementary information changes accept.pdf]

Supplementary data

Supplementary figure 1

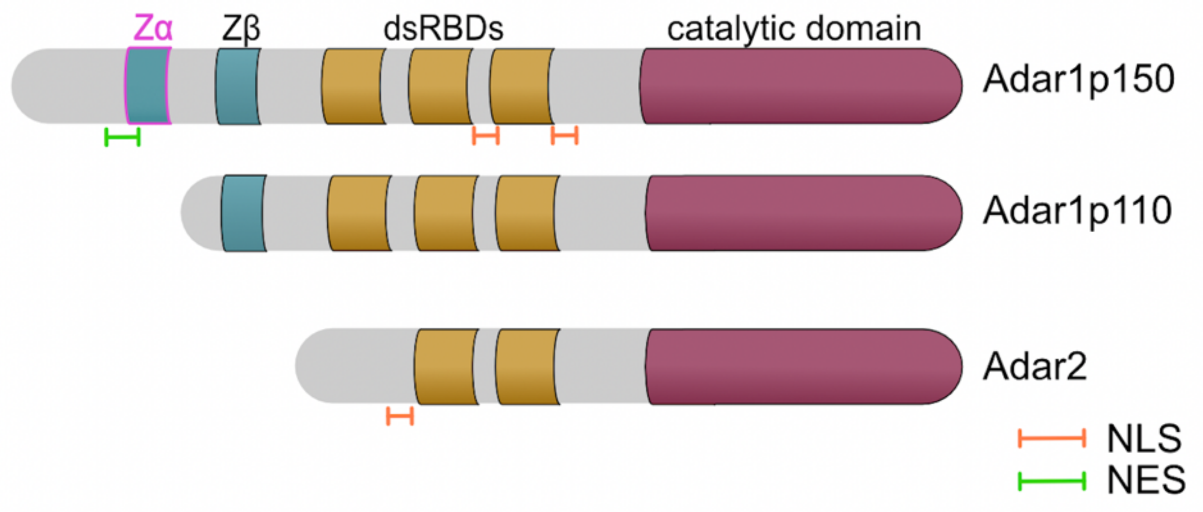

Supplementary Figure 1: Domain organization of active mammalian ADAR proteins
ADARs contain a conserved C-terminal catalytic-deaminase domain and two or three double-stranded RNA binding domains (dsRBDs). Whilst both ADAR1 isoforms have Z-DNA binding domain β (Zβ), full-length ADAR1p150 isoform bears an additional N-terminal Z-DNA binding domain α (Zα) and a nuclear export signal (NES). All ADAR variants possess nuclear localization signals (NLS). While the NLS of ADAR2 is localized at the N-terminus, the NLS of ADAR1 is bimodular assembled around the third dsRBD.

Supplementary figure 2

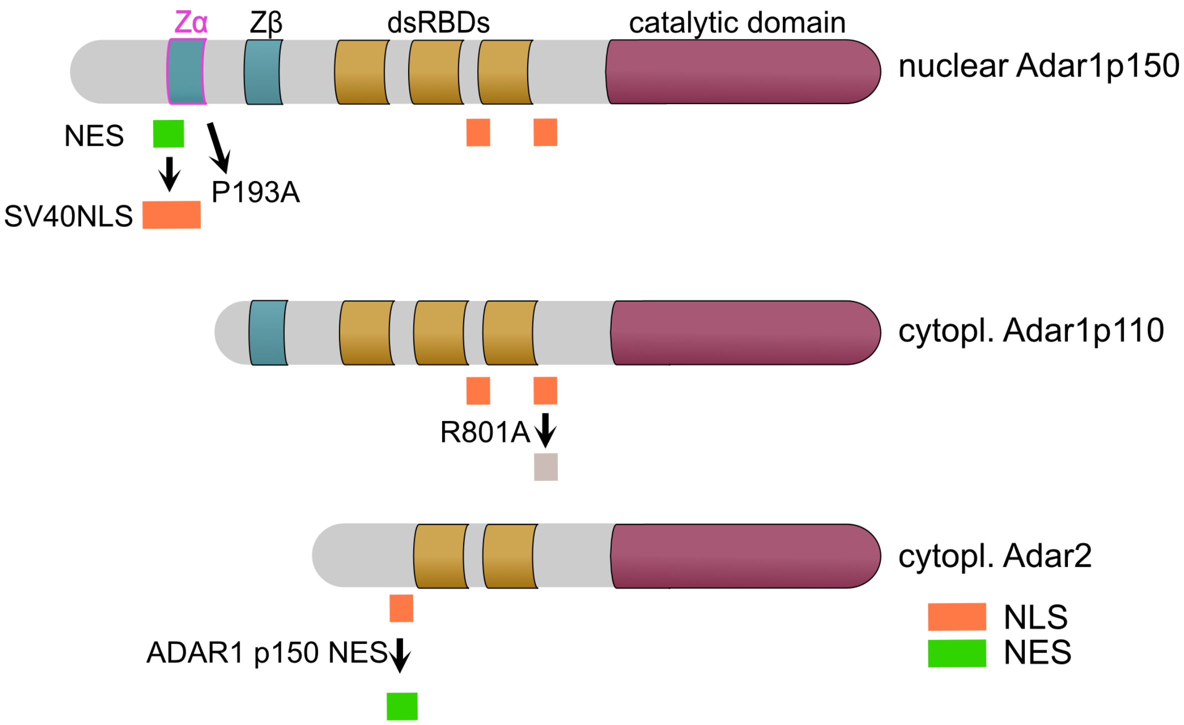

Supplementary figure 2: Schematic representation of constructs made to generate mislocalized and ZBDα mutant ADARs

To construct nuclear ADAR1p150 the entire NES (CLSSHFQELSIYQDQEQRILKFLEEL, aa 125-150) was deleted and replaced with a SV-40 NLS (PKKKRKVEDP). The ZBDα mutant was generated by exchanging P193 to A193 to mimic the frequent mutation identified in AGS patients. Cytoplasmic ADAR1p110 was generated by introducing a point mutation: R801A that abolishes NLS activity. To prepare cytoplasmic ADAR2, the N-terminus was deleted (1-72) and replaced with the minimal NES of ADAR1p150 (CLSSHFQELSIY, 125-136)

Supplementary figure 3

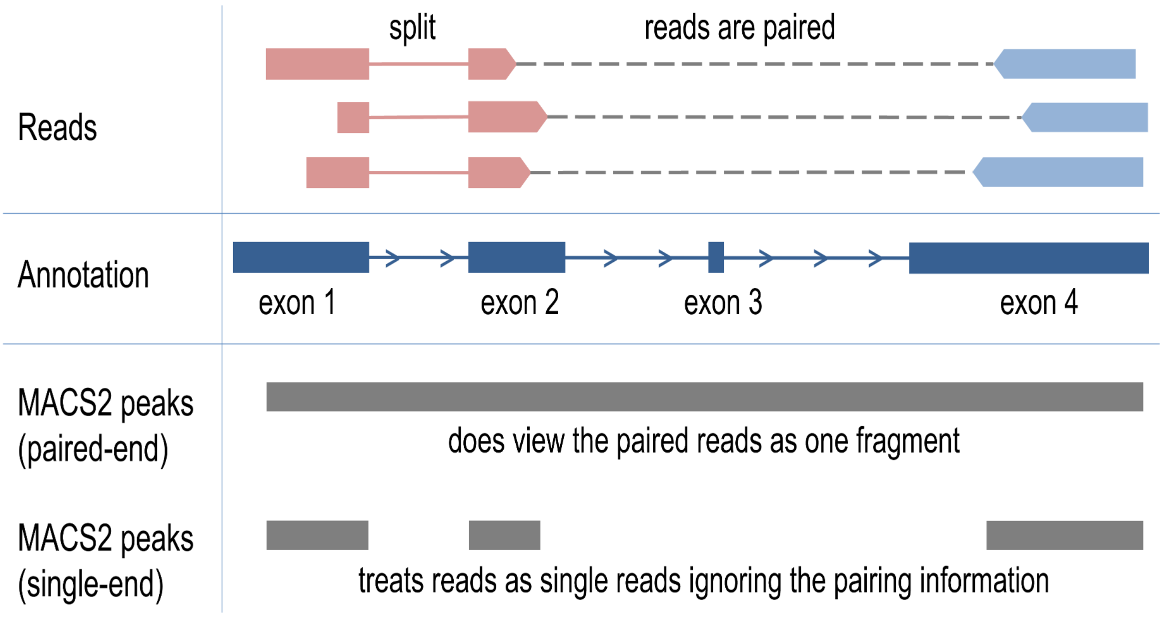

Supplementary figure 3: Mapping of paired-end reads across introns

Paired reads are viewed as one fragment in MACS2 and thus produce very long peaks which include also regions with no read. First row depicts example reads, second an annotion, third the peaks called by MACS2.

Supplementary figure 4

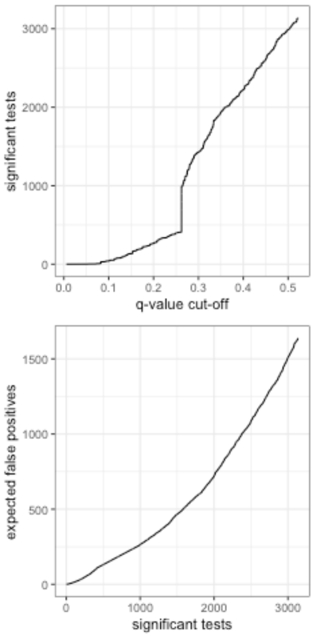

Supplementary Figure 4: Plot depicting the q values versus the number of significant tests (top); (bottom) the expected number of false positives versus the total number of significant tests given by the q values (bottom).

Supplementary figure 5

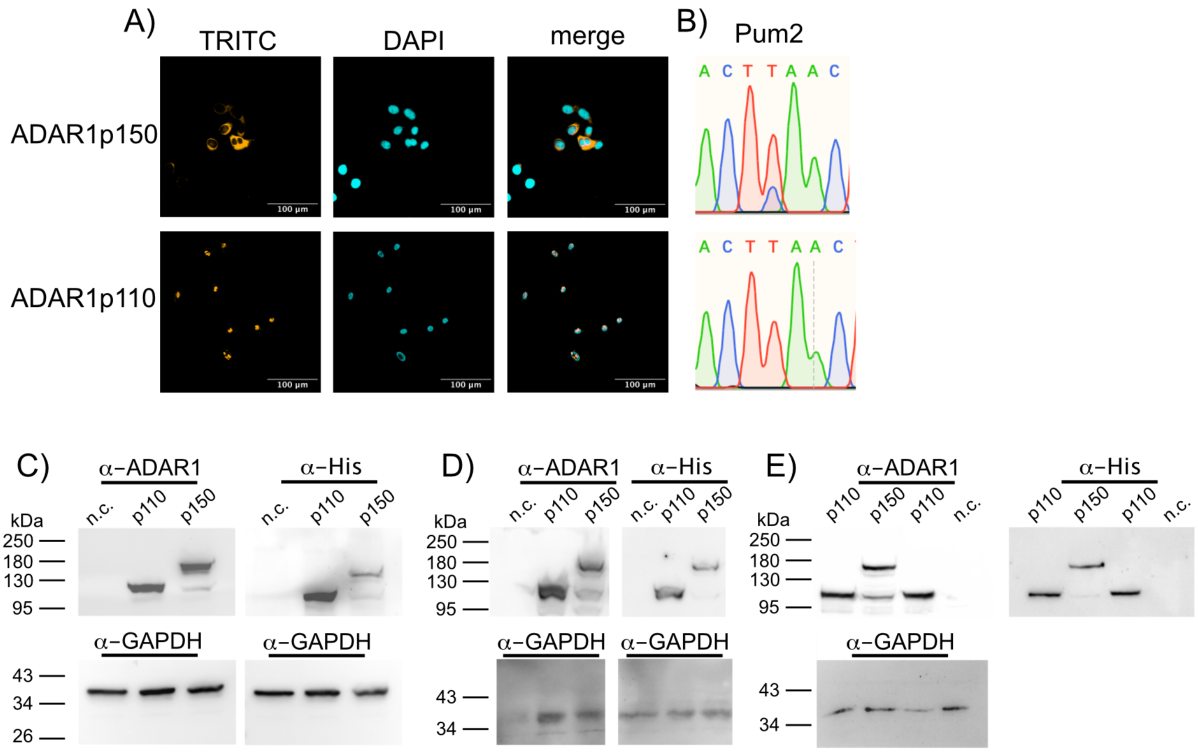

Supplementary figure 5: Over-expressed ADAR1-isoforms showed typical cellular localization and distinct editing patterns.

A) ADAR1p150 is mainly localized to the cytoplasm, whereas ADAR1p110 is primarily nuclear. TRITC channel shows transfected constructs in confocal sections, and nuclear DNA is stained with DAPI (Scale bar: 100 µm). B) Editing site in the 3'UTR of Pum2 (chr12: 8750269) is edited by ADAR1p150 but not by ADAR1p110. The reverse strand is sequenced. Consequently, an A to I event is seen as a T to C conversion in the chromatogram. C), D), E) Three independent western blots showing the expression of human tagged ADAR1p150 and ADAR1p110 in transfected MEF cells. Western blot was detected with a rat polyclonal serum against human ADAR1 (α–ADAR1) or an antibody against a C-terminal His-tag (α–His). To demonstrate equal loading, the lower part of the blot was also detected with an antibody against GAPDH (α–GAPDH).

Supplementary figure 6

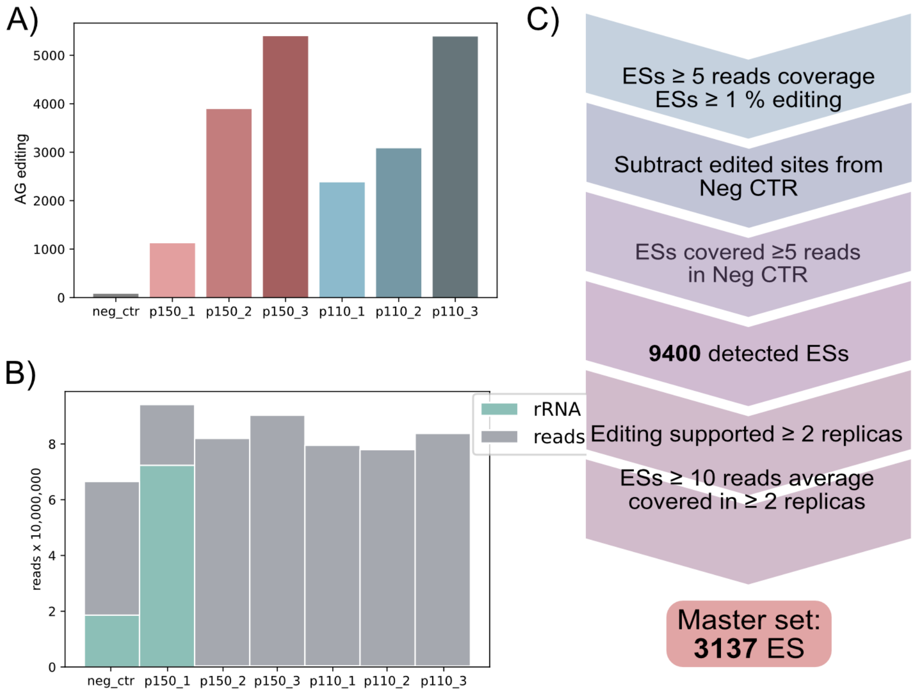

Supplementary figure 6: Restoring ADAR1 expression in editing-deficient cells produces an authentic set of editing sites

A) Editing at known-editing sites detected in each sample. Only few A to G transitions were detected in the negative control: 78 sites. Editing rises dramatically upon transfection with any ADAR1-isoform, ranging from 1.121 to 5.395 detected sites. Sites covered ≥ 5 reads and showing editing rates ≥ 0,01. B) rRNA reads in each sample (depicted in green). The first replica of ADAR1p150 shows a fraction of rRNA reads (77 %). While the negative control also has a prominent rRNA fraction the remaining samples exhibit a minimal rRNA portion (less than 1 %). C) Filtering strategy for the master set generation. Sufficiently edited (≥ 1%) and covered (≥ 5 reads) editing sites were collected for each sample. Next, detected edited editing sites in negative control were subtracted from each sample. Each detected edited editing site was covered ≥ 5 reads in negative control. All edited editing sites passing above criteria were combined, resulting in 9.400 sites. Next, sites covered by at least 5 reads and showing an editing ratio of at least 1% in 2 out of 3 replicas were collected. Finally, only sites with sufficient coverage in datasets of both isoforms (≥ 10 reads in average, covered in ≥ 2 replicas) formed the final 'master' dataset containing 3.137 editing sites.

Supplementary figure 7

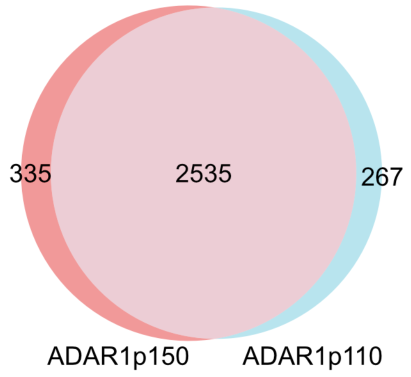

Supplementary figure 7: ADAR1 isoforms have a large overlap of editing sites

The majority of sites can be edited by both isoforms (also considering a minor editing rate below 1%). Still, 335 and 267 sites are exclusively edited by ADAR1p150 or ADAR1p110 in our dataset.

Supplementary Figure 8

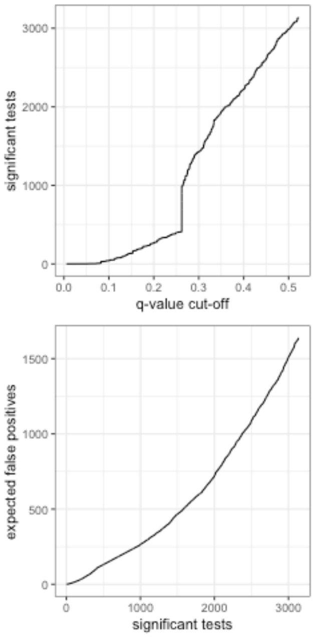

Supplementary Figure 8: Plot depicting the q values versus the number of significant tests (top); and the expected number of false positives versus the total number of significant tests given by the q values (bottom).

Supplementary figure 9

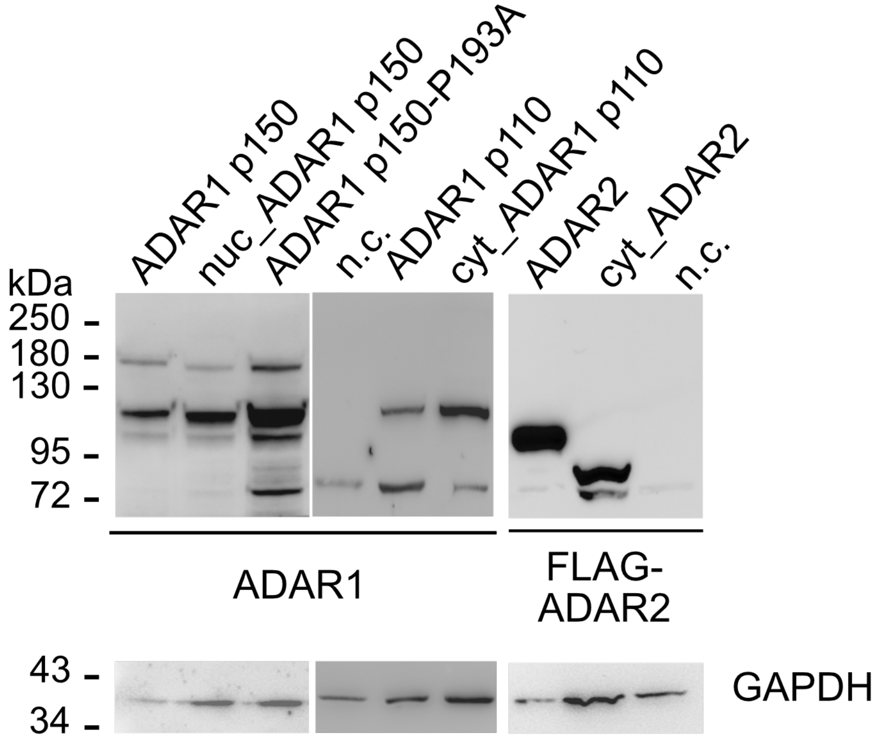

Supplementary figure 9. Expression of mutated ADAR1 versions upon transfection detected by western blotting. MEF cells were transfected with plasmids encoding the indicated protein versions. Cell lysates were separated on SDS PAGE gels and detected with an anti-ADAR1 antibody or a FLAG-ADAR2 antibody as indicated. As a loading control, the lower part of the gel was detected with a GAPDH antibody.

Supplementary figure 10

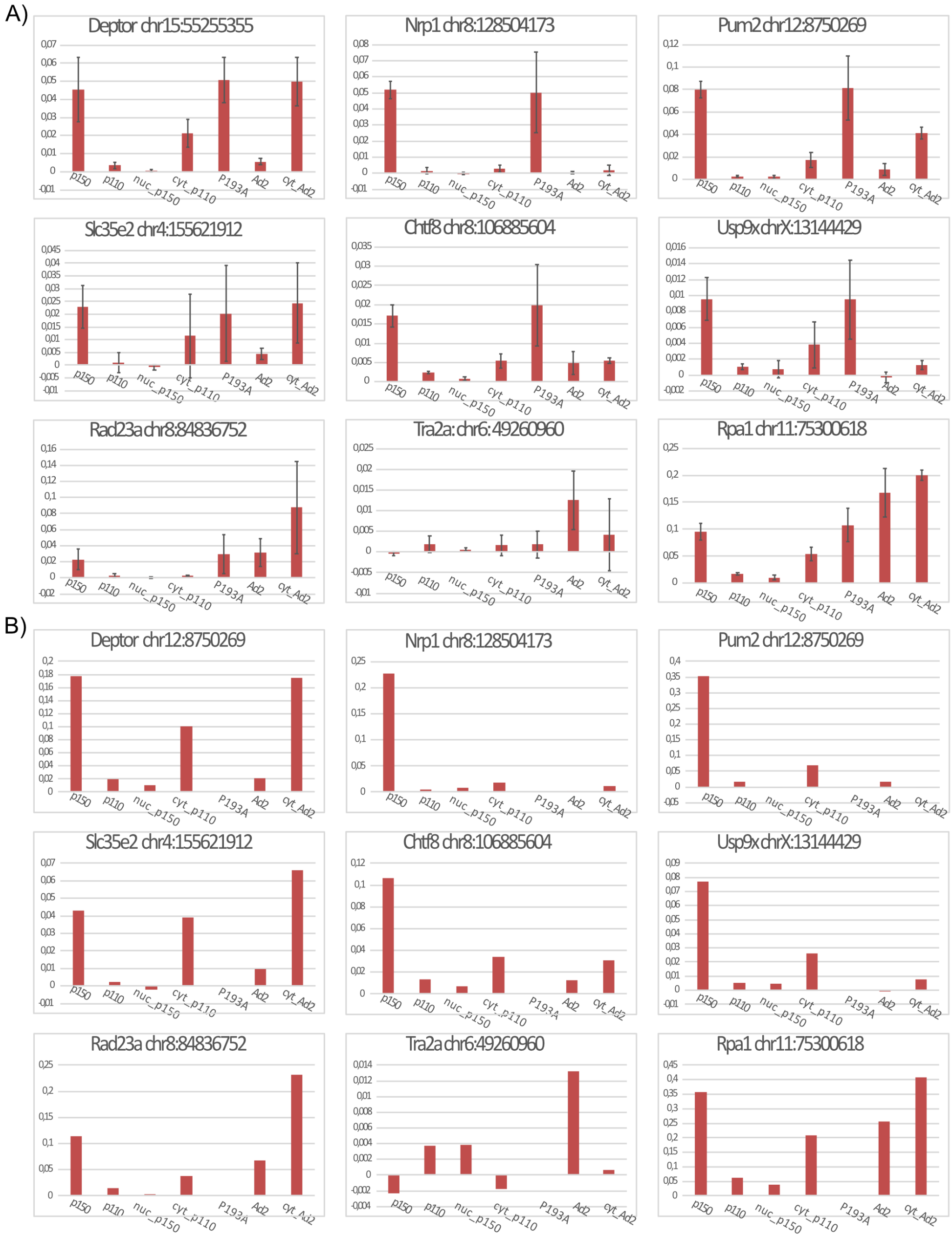

Supplementary figure 10: Editing levels determined by amplicon-seq in ADAR1p150-targets The overall editing pattern remains similar upon transfection of 5µg (A) and 10 µg (B) of plasmid Overall, substrates edited by ADAR1p150 are also well edited by ADAR1p150 carrying a mutation in ZBDa but also by cytoplasmic ADAR2. In contrast, nuclear ADAR versions edit those sites with reduced efficiency. (B) Editing in the cells transfected with 10 µg of DNA does not contain mZ – ADAR1p150 P193A.

Supplementary figure 11

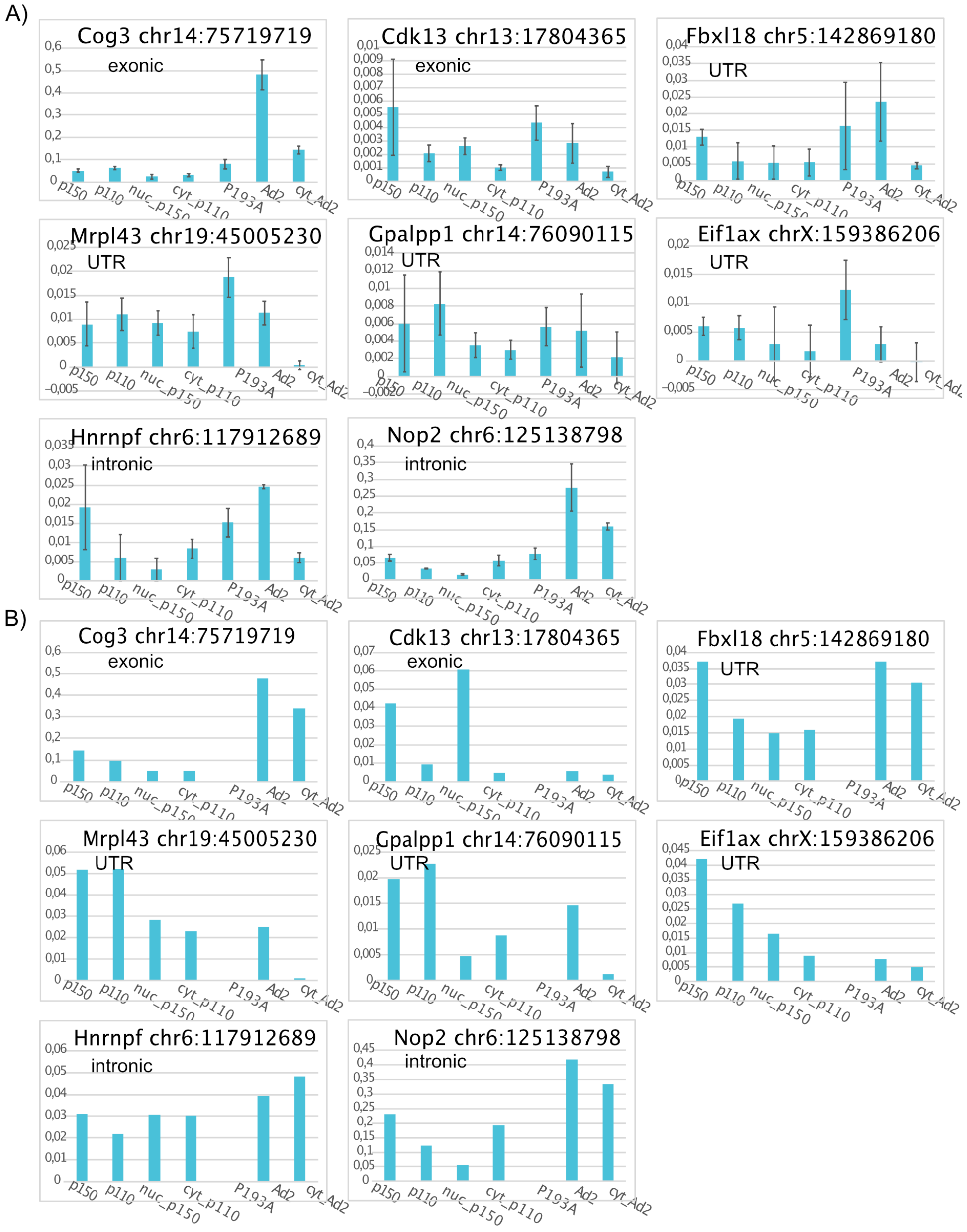

Supplementary figure 11: Editing levels determined by amplicon-seq at selected ADAR1p110-targets

a) Sites edited by ADAR1p110 are also well edited by ADAR2 but also by cytoplasmic ADAR p150. Overall, these sites show less preference for a given ADAR ioform. This may be the result of the nucleo-cytoplasmic huttling of ADAR1p150. The editing pattern remains similar upon transfection of 5μg and 10 μg of plasmid DNA. A) Editing in the cells transfected with 5 μg of DNA; the experiment was conducted in triplicates; B) Editing in the cells transfected with 10 μg of DNA (this experiment does not contain mZ – ADAR1p150 P193A).

Supplementary figure 12

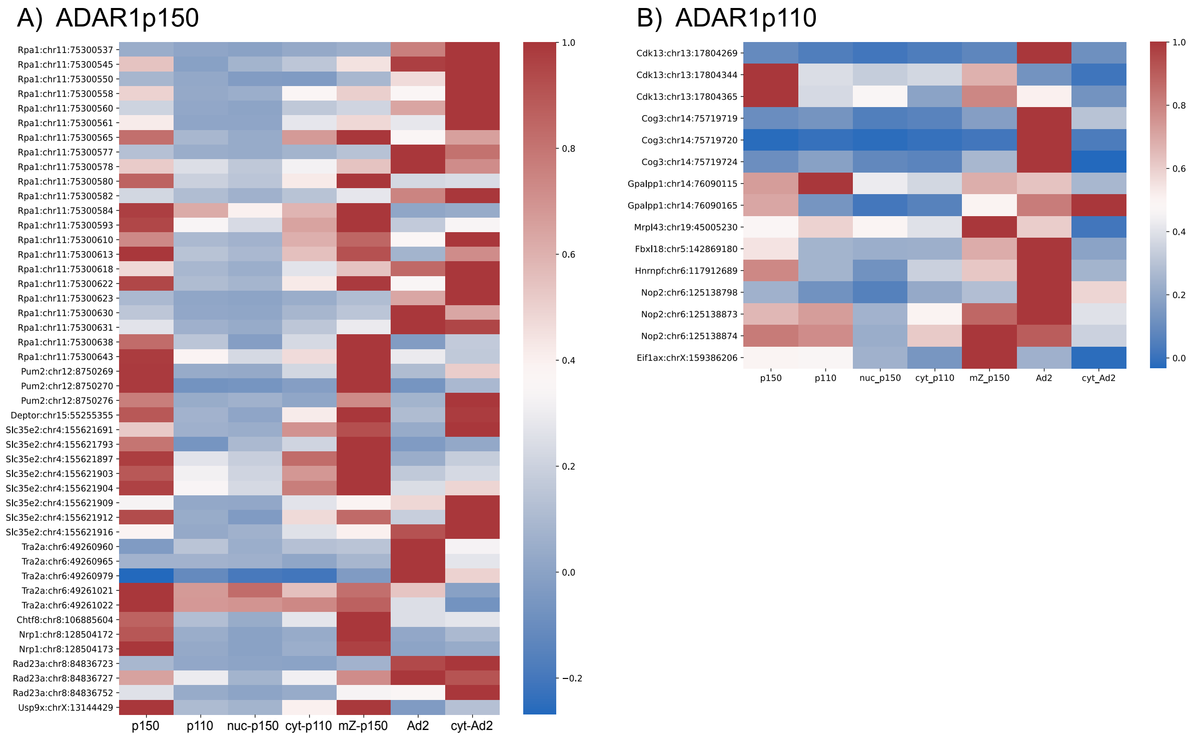

Supplementary figure 12: Heat maps of normalized editing ratios of all editing sites identified by amplicon-seq that are edited at levels ≥ 0,5%

In the regions selected for amplicon seq several closely spaced editing sites are detected. Surprisingly, some very close sites show dramatically distinct editing patterns. A) Editing targets selected as preferentially edited by ADAR1p150 based on the ADAR1-editome obtained in MEFs. These sites can also be edited by cytoplasmic ADAR1 p110 or cytoplasmic ADAR2 and are not affected by a mutation in ZBDα B) Editing targets selected as preferentially edited by ADAR1p110 based on the ADAR1-editome in MEFs are also edited by ADAR2 Interestingly, many of these sites can also be edited by ADAR p150 in this experiment.

Supplementary figure 13

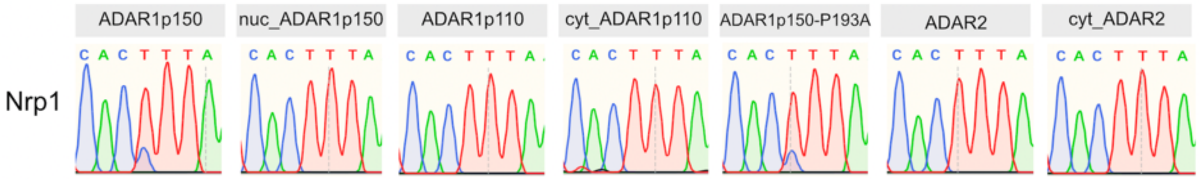

Supplementary figure 13: Sanger sequencing validates the Nrp1 editing pattern for wild-type and mutant ADAR isoforms as identified by amplicon-seq. The reverse strand is sequenced. Consequently, an A to I event is seen as a T to C conversion in the chromatogram.

Supplementary figure 14

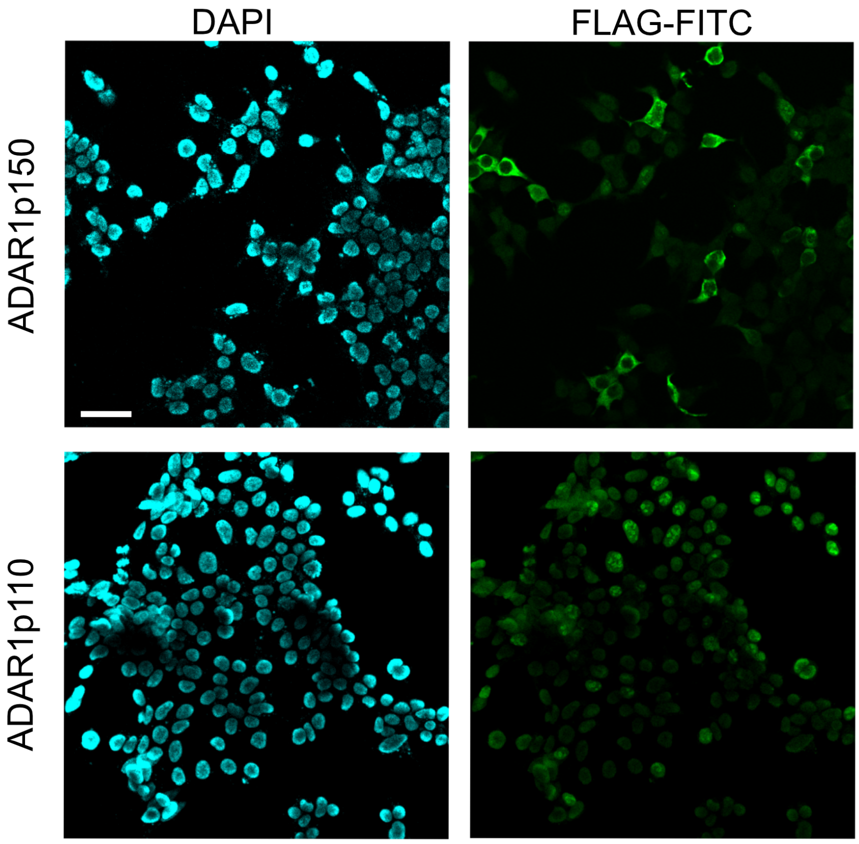

Supplementary figure 14: Immunofluorescence of HEK293 cells transfected with FLAG-tagged human ADAR1p150 (top) and ADAR1p10 (bottom). Transfected ADAR versions were deteced by immunofluorescence with an anti FLAG antibody. As expected, ADAR1p150 localizes to the cytoplasm while ADAR1p110 is localized to the nucleus. Scale bar=50µm.

Supplementary figure 15

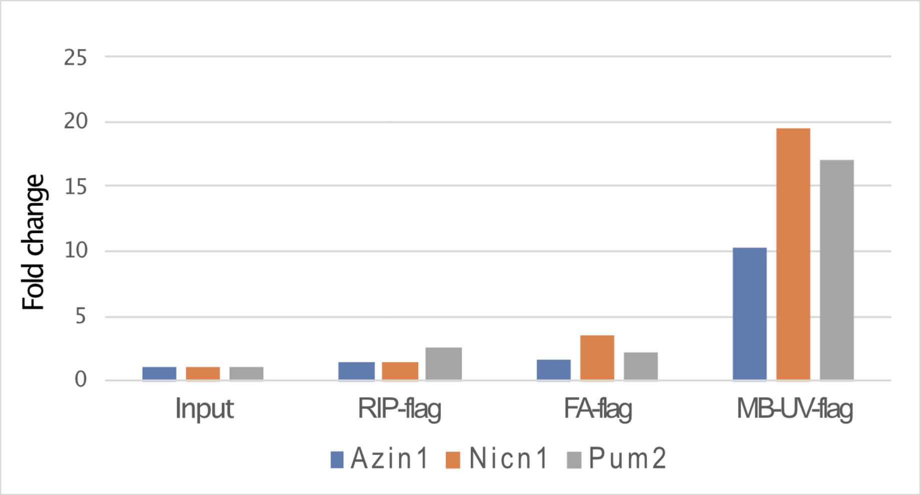

Supplementary figure 15: qPCR evaluation of ADAR1 targets in IP and corresponding input fraction upon selected cross-linking conditions.

RIP-flag: no cross-linking applied; FA-flag: formaldehyde cross-linking; MB+UV-flag: a combination of methylene blue and ultra-violet light 254 nm. The experiment was conducted with the ADAR1p150 isoform. Fold-change enrichment is normalized to the corresponding input.

Supplementary figure 16

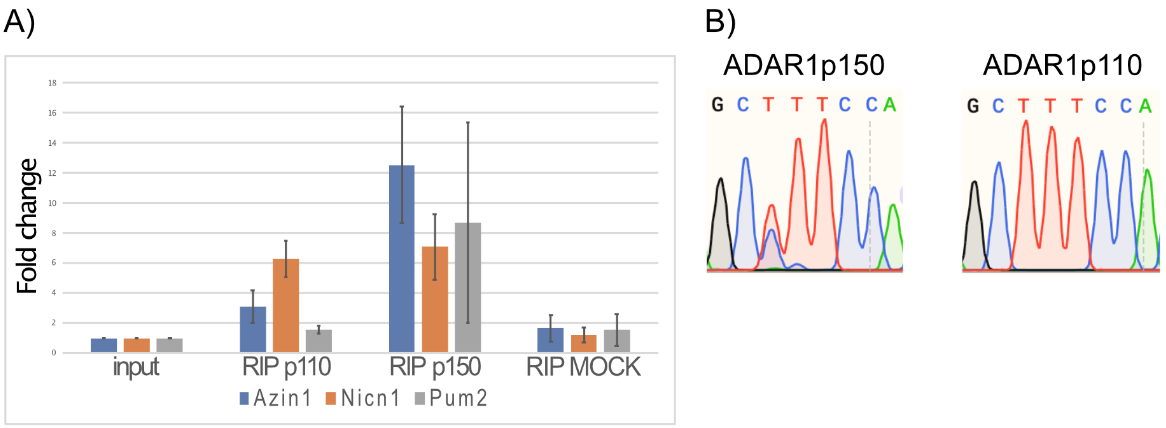

Supplementary figure 16: Verification of substrate enrichment and editing upon ADAR1 isoform IP

A) qPCR evaluation of enrichment of ADAR1-targets in IP-fractions. The enrichment was normalized to the relevant input sample. RIP using p110 and p150 were conducted in triplicates and RIP with mock transfection in duplicate.

B) Sanger sequencing traces of a selected editing site. Azin1 (chr8:103841636) is primarily edited by ADAR1p150. The reverse strand is sequenced. Consequently, an A to I event is seen as a T to C conversion in the chromatogram-indicated with an asterisk (*)

Supplementary figure 17

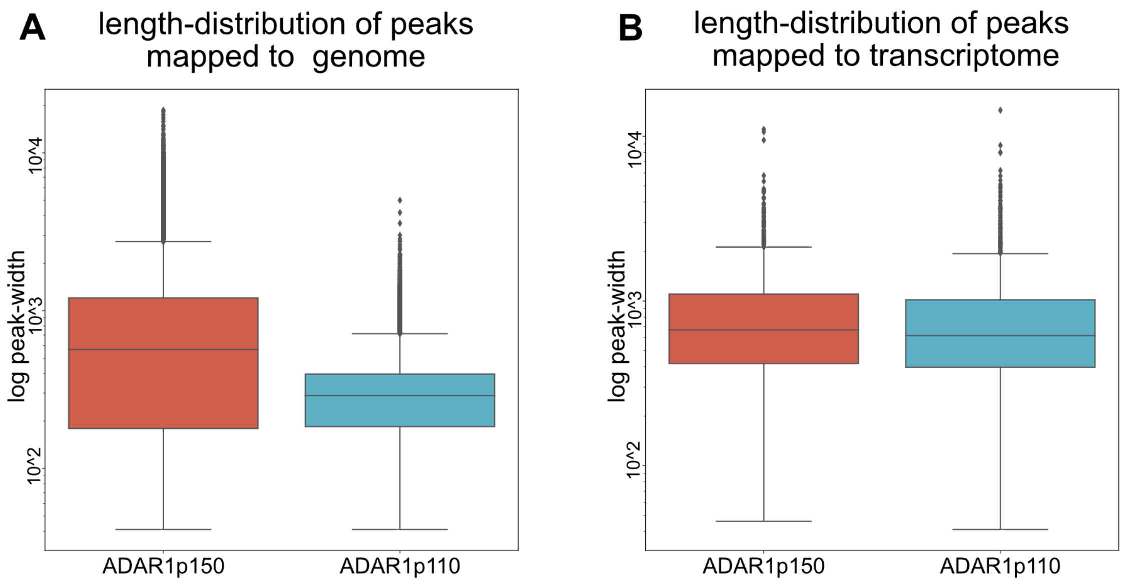

Supplementary figure 17: Peak-width distribution in RIP-seq experiments mapped to genome or transcriptome

A) Peak-length based on genomic coordinates, ADAR1p150-peaks median: 951 nt and ADAR1p110-peaks median: 328 nt), y-axis is in log scale. B) Peak-length based on transcriptomic coordinates, ADAR1p150-peaks median: 885 nt and ADAR1p110-peaks median: 855 nt), y-axis is in log scale.

Supplementary figure 18

A) Azin1 - chr15: 38491612, 38491613

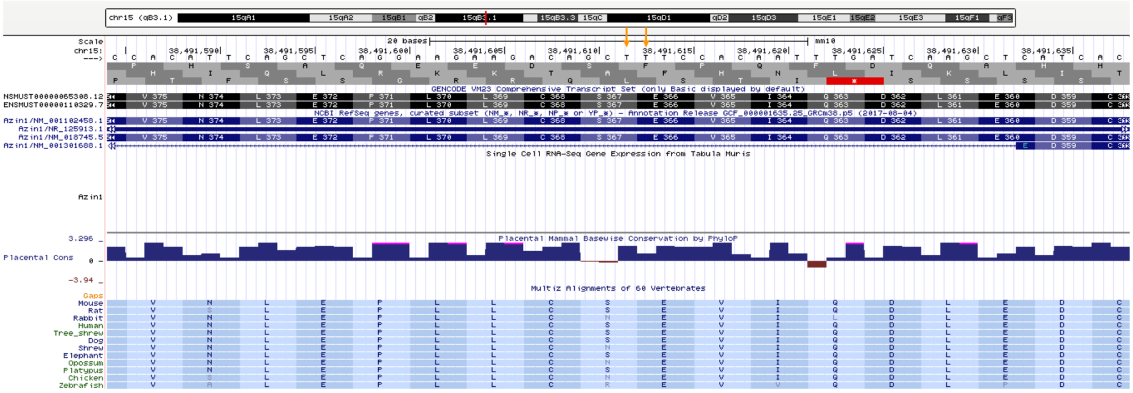

B) Nrp1 - 3'UTR, chr8:128504172, 128504173

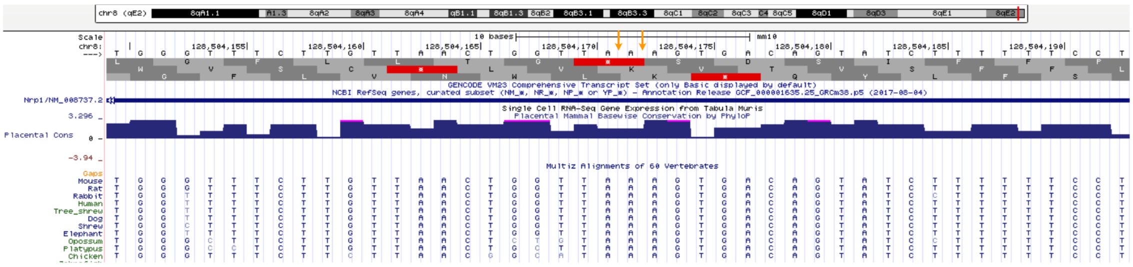

Supplementary figure 18: Genome browser view (http://genome.ucsc.edu) for selected editing sites demonstrating conservation amongst pacentaria. A) A conserved protein recoding editing site in Azin1 - chr15: 38491612, 38491613; B) The editing site in the 3' UTR of Nrp1 seems similarly conserved, chr8:128504172, 128504173.

Supplementary figure 19

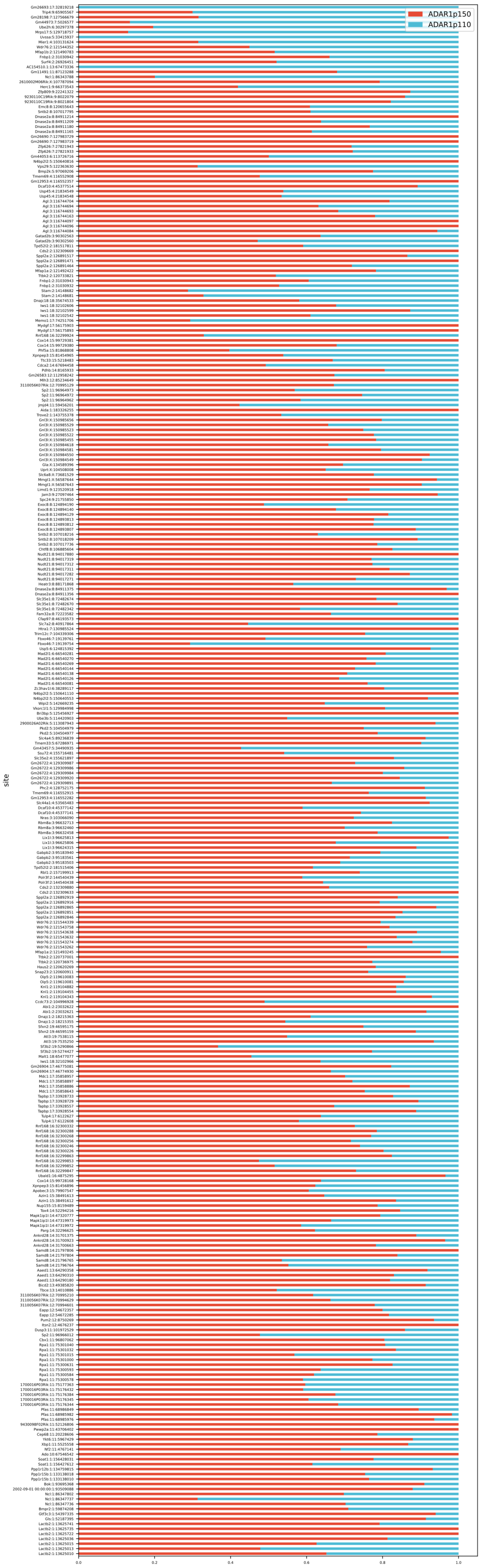

Supplementary figure 19: ADAR1p150 and ADAR1p110-mediated editing ratios

Sites found edited by ADAR1p150 in the thymus by (Kim et al., 2021 (1))were tested for their enzyme preference in our experiments performed in MEFs. Red bars: edited by ADAR1 p150. Turquoise bars: edited by ADAR1 p110.

Supplementary figure 20

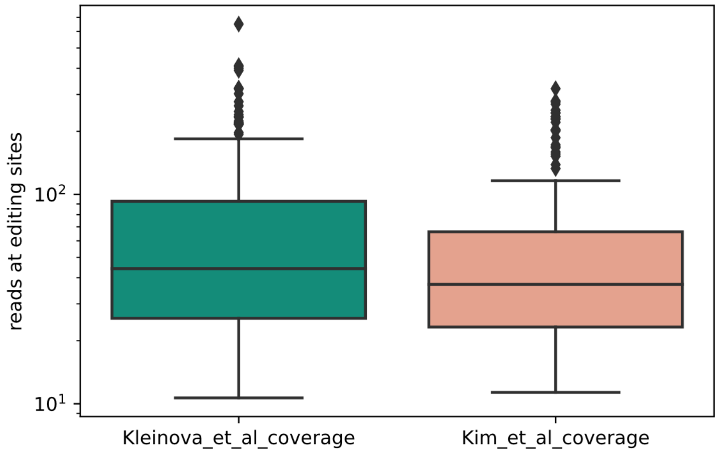

Supplementary figure 20: Read-coverage comparison between the MEFs-editome experiment (this study) and ADAR1p150-specific sites identified by Kim et al., (1). Coverage of all overlapping sites identified in the brain (green, this study) and the thymus (light brown, Kim et al., 2021 (4).

Supplementary figure 21

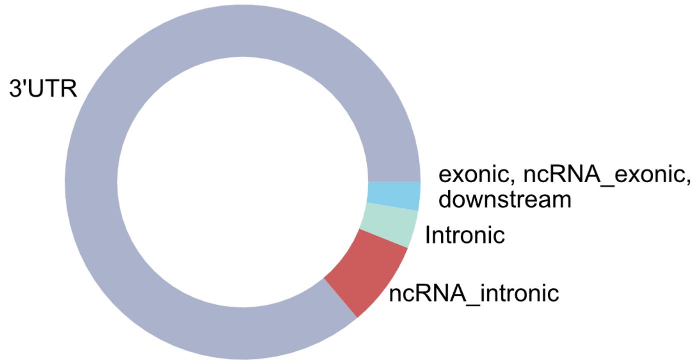

Supplementary figure 21: localization of overlapping ADAR1 p150 sites found in this study and those identified by Kim et al., (1).
The majority of the overlapping sites between p150 specific ES extracted from Kim et al. and the ADAR1 editome identified in this study localize to 3' UTRs.

1. Kim, J.I., Nakahama, T., Yamasaki, R., Costa Cruz, P.H., Vongpipatana, T., Inoue, M., Kanou, N., Xing, Y., Todo, H., Shibuya, T. *et al.* (2021) RNA editing at a limited number of sites is sufficient to prevent MDA5 activation in the mouse brain. *PLoS Genet*, **17**, e1009516.
